# p62 Promotes Survival and Hepatocarcinogenesis in Mice with Liver-Specific NEMO Ablation

**DOI:** 10.3390/cancers14102436

**Published:** 2022-05-15

**Authors:** Vangelis Kondylis, Farina Schneider, Fabian Schorn, Nikos Oikonomou, Beate Katharina Straub, Sabine Werner, Philip Rosenstiel, Manolis Pasparakis

**Affiliations:** 1 Institute for Genetics, University of Cologne, D-50674 Cologne, Germany; noikonom@uni-koeln.de (N.O.); pasparakis@uni-koeln.de (M.P.); 2 Cologne Excellence Cluster on Cellular Stress Responses in Aging-Associated Diseases (CECAD), University of Cologne, D-50931 Cologne, Germany; farinaschneidercosentino@googlemail.com (F.S.); fabian.schorn@uk-koeln.de (F.S.); 3 Center for Molecular Medicine (CMMC), University of Cologne, D-50931 Cologne, Germany; 4 Institute of Pathology, Faculty of Medicine, University Hospital Cologne, D-50931 Cologne, Germany; 5 Institute of Pathology, University Hospital Mainz, D-55131 Mainz, Germany; beate.straub@unimedizin-mainz.de; 6 Institute of Molecular Health Sciences, Department of Biology, ETH Zurich, 8093 Zurich, Switzerland; sabine.werner@biol.ethz.ch; 7 Institute of Clinical Molecular Biology, Christian Albrecht University of Kiel, Schleswig-Holstein University Hospital, D-24105 Kiel, Germany; p.rosenstiel@mucosa.de

**Keywords:** IκB kinase (IKK) complex, autophagy, chronic liver disease, liver injury, hepatocarcinogenesis, Keap1-Nrf2 activation, genetic mouse models

## Abstract

**Simple Summary:**

Chronic liver injury is a predisposing factor for hepatocellular carcinoma (HCC) development. p62-mediated Nrf2 overactivation has been shown to drive liver injury and HCC in mice with hepatic impairment of autophagy. Here, we addressed the role of this pathway in a liver disease mouse model that does not exhibit inherent autophagy defect. Genetically-induced Nrf2 overactivation without concomitant strong increase in p62 expression did not aggravate liver injury and hepatocarcinogenesis. In contrast, p62-driven Nrf2 overactivation was prominent in liver tumors of mice that expressed a p62 mutant and showed enhanced hepatocarcinogenesis. Moreover, a negative correlation was observed between p62/Nrf2^high^ liver tumors and the autophagosome marker LC3, suggesting that acquired autophagy defects precede the activation of this pro-tumorigenic pathway. Our results suggest that autophagy activators or Nrf2 inhibitors could be considered therapeutically in cases of p62/Nrf2^high^ liver tumors.

**Abstract:**

SQSTM1/p62 is a multitasking protein that functions as an autophagy receptor, but also as a signaling hub regulating diverse cellular pathways. p62 accumulation in mice with autophagy-deficient hepatocytes mediates liver damage and hepatocarcinogenesis through Nrf2 overactivation, yet the role of the p62-Keap1-Nrf2 axis in cell death and hepatocarcinogenesis in the absence of underlying autophagy defects is less clear. Here, we addressed the role of p62 and Nrf2 activation in a chronic liver disease model, namely mice with liver parenchymal cell-specific knockout of NEMO (NEMO^LPC-KO^), in which we demonstrate that they show no inherent autophagy impairment. Unexpectedly, systemic p62 ablation aggravated the phenotype and caused early postnatal lethality in NEMO^LPC-KO^ mice. Expression of a p62 mutant (p62ΔEx2-5), which retains the ability to form aggregates and activate Nrf2 signaling, did not cause early lethality, but exacerbated hepatocarcinogenesis in these mice. Our immunohistological and molecular analyses showed that the increased tumor burden was only consistent with increased expression/stability of p62ΔEx2-5 driving Nrf2 hyperactivation, but not with other protumorigenic functions of p62, such as mTOR activation, cMYC upregulation or increased fibrosis. Surprisingly, forced activation of Nrf2 per se did not increase liver injury or tumor burden in NEMO^LPC-KO^ mice, suggesting that autophagy impairment is a necessary prerequisite to unleash the Nrf2 oncogenic potential in mice with autophagy-competent hepatocytes.

## 1. Introduction

Hepatocyte death is an early event in chronic liver disease (CLD), such as alcoholic and non-alcoholic steatohepatitis (ASH and NASH) and viral hepatitis. Prolonged liver injury promotes CLD progression by triggering liver regeneration, inflammation and fibrosis, a setting that eventually leads to cirrhosis and accumulation of oncogenic mutations, thereby predisposing the development of hepatocellular carcinoma (HCC), which is the most common type of liver cancer. Conversely, evasion of cell death during hepatocarcinogenesis promotes survival of premalignant hepatocytes and HCC establishment. Targeting cell death in CLD patients has been an attractive therapeutic approach, hence understanding the exact molecular mechanisms underlying liver damage is of great importance [1,2,3].

Mice with liver parenchymal cell (LPC)-specific knockout of NF-kappa-B essential modulator (NEMO)/IKKγ (NEMO^LPC-KO^) constitute a genetic mouse model that recapitulates the main features of CLD. NEMO^LPC-KO^ mice exhibit spontaneous chronic liver damage that leads to compensatory hepatocyte proliferation, activation of hepatic progenitor cells, hepatitis, fibrosis and HCC development [4,5]. NEMO is the regulatory subunit of the IκB kinase (IKK) complex, which also comprises the IKK1/IKKα and IKK2/IKKβ kinases. The IKK complex is essential for NF-κB activation, but also exerts NF-κB-independent pro-survival functions [4,6,7]. More specifically, NEMO prevents hepatocyte death by inhibiting receptor-interacting protein kinase 1 (RIPK1) kinase-dependent activation of FADD/Caspase-8-mediated apoptosis [4]. However, the upstream pathways triggering liver damage in NEMO^LPC-KO^ mice remain unclear, as deletion of the extrinsic cell death receptors TNFR1, TRAIL-R and Fas/CD95 in LPCs did not prevent cell death [8].

IKKs have been implicated in autophagy induction in human and murine cancer cells [9] and in transcription of autophagy genes in mouse embryonic fibroblasts in an NF-κB-independent way [10]. Other studies, however, have reported that IKK signaling negatively regulates autophagy [11]. For example, under conditions of constitutively active Akt, IKKα was shown to activate the mechanistic target of rapamycin (mTOR), which suppresses autophagy [12]. Mice lacking IKKβ in hepatocytes (IKKβ^Δhepa^) showed impaired starvation-induced autophagosome formation in the liver, supporting that IKK regulates autophagy in vivo [9]. Moreover, ablation of the IKK upstream regulator TGFβ-activated kinase 1 (TAK1) was also shown to impair autophagy in hepatocytes [13]. This effect was attributed to lack of TAK1-mediated activation of AMP-activated protein kinase (AMPK) and mTOR inhibition, both upstream events that inhibit autophagy [14]. Interestingly, deletion of IKKα, but not IKKβ, from pancreatic acinar cells led to spontaneous pancreatitis development, an effect associated with defects in autophagic degradation [15]. Together, these data highlight a potential tissue- and context-specific impact of the IKK complex subunits in regulating autophagy.

Impaired autophagy has been associated with CLD pathogenesis in mouse models and humans with ASH and NASH [16,17]. Similar to NEMO^LPC-KO^ mice, mice with hepatocyte-specific deletion of ATG5 or ATG7 (ATG5^Δhepa^, ATG7^Δhepa^) develop spontaneous liver damage, inflammation, fibrosis, hepatomegaly and liver tumors [18,19,20]. A typical feature of autophagy-impaired hepatocytes is the accumulation of p62/SQSTM1, an autophagy receptor and itself an autophagy substrate, in cytoplasmic aggregates [21]. These aggregates (also known as Mallory-Denk and hyaline bodies) have been associated to autophagy defects during CLD progression in humans [16,22]. Interestingly, although p62 usually protects cells from death induction [23], its loss significantly ameliorated the liver pathology and tumorigenesis observed in ATG7^Δhepa^ mice [18,21].

In addition to its well-described role as an autophagy receptor, p62 is a multifaceted protein that acts as a signaling hub through various molecular interactions. Cellular pathways that affect cell survival and tumorigenesis, such as NF-κB, mTORC1 and the nuclear factor erythroid 2-related factor 2 (Nrf2/Nfe2l2), are shown to be regulated by p62 [24,25]. Out of all p62 functions, p62-mediated overactivation of Nrf2 through the sequestration of Kelch-like ECH-associated protein 1 (Keap1) appears to be critical for liver damage and tumorigenesis in mice with autophagy-deficient hepatocytes, as Nrf2 ablation was shown to rescue these pathological parameters [20,26]. However, while a contribution of the Nrf2 activation to hepatocarcinogenesis has been established by human HCC whole-exome sequencing studies [27,28,29], its mechanistic contribution to hepatocyte death remains paradoxical, as Nrf2 activation normally promotes cell survival under oxidative and metabolic stress conditions [30].

In this study, we first assessed whether impaired autophagy could be the cell death trigger in NEMO-deficient hepatocytes, and we compared the phenotypes of NEMO^LPC-KO^ and ATG16L1^LPC-KO^ mice, the latter exhibiting LPC-specific ablation of the gene encoding the essential for autophagy protein ATG16L1. Based on our results, we then used NEMO^LPC-KO^ mice as a model of chronic liver disease with no intrinsic autophagy impairment to address the role of p62 and Nrf2 activation per se in liver injury and hepatocarcinogenesis. Finally, to examine the relationship between p62 expression, Nrf2 hyperactivation and potentially acquired autophagy defects during tumorigenesis in NEMO^LPC-KO^ mice, we performed correlative analyses of relevant markers in (pre)neoplastic lesions.

## 2. Materials and Methods

### 2.1. Mice

The following mouse lines were used: *Nemo*^FL^ [31], *Ikk2*^FL^ [32], *Ikk1*^FL^ [33], *R26Ikk2ca*^sFL^ [34], *Atg16l1*^FL^ [35], *p62*^ko^ [36], *p62*^FL^ (EUCOMM) [37], *GFP-LC3*^tg^ [38], and *Nrf2ca*^sFL^ [39]. All alleles were maintained on a C57BL/6 genetic background. Mice were crossed to Alfp-cre transgenic mice [40] to generate liver parenchymal cell-specific knockouts or expression of the respective mutants. Littermates carrying the floxed alleles but not the *Alfp-Cre* transgene served as controls. Mice were housed in individually ventilated cages at 22 °C (±2 °C) in specific pathogen-free (SPF) mouse facilities at the Institute for Genetics and the CECAD Research Centre, University of Cologne, under a 12 h light cycle, and were given a regular chow diet (Harlan, diet no. 2918) and water ad libitum. The microbiological status was examined as recommended by the Federation of European Laboratory Animal Science Associations (FELASA), and the mice were free of all listed pathogens. All animal procedures were conducted in accordance with European, national and institutional guidelines and protocols, and were approved by local government authorities (Landesamt für Natur, Umwelt und Verbraucherschutz Nordrhein-Westfalen, Recklinghausen, Germany) under the license number 84-02.04.2016.A452. Animals requiring medical attention were provided with appropriate care and were sacrificed when reaching pre-determined criteria of disease severity. No other exclusion criteria existed. Comparable numbers of female and male mice of the indicated genotypes were randomly analyzed.

### 2.2. Hepatocyte Isolation and Culture

Primary hepatocytes were isolated from 4-to-5-week-old mice as described previously [41]. In brief, mice were perfused via the vena cava with solution I (EBSS without Ca^2+^ and Mg^2+^, supplemented with 0.5 mM EGTA). Subsequently, perfusion with 50 mL of collagenase solution (EBSS with Ca^2+^ and Mg^2+^, 10 mM HEPES, 3810 U collagenase and 2 mg Trypsin inhibitor) was performed, and single cell suspensions of perfused liver were generated using a 70 µm nylon mesh. Hepatocytes were washed twice in high glucose DMEM supplemented with 1.5% FCS, penicillin and streptomycin, followed by seeding on collagen-coated plates or fibronectin-coated glass coverslips. The medium was renewed 4 hr later to remove any unattached/dying cells. Primary hepatocytes were treated the day after isolation and plating for the indicated times using the following reagent concentrations: 5 μg/mL rapamycin (LC Laboratories), 150 nM bafilomycin A1 (Sigma-Aldrich, Schnelldorf, Germany), 25 μM CCCP (Sigma-Aldrich), 5 mM 3-MA (Sigma-Aldrich). The in vitro experiments in NEMO-deficient and respective control hepatocytes were performed in the presence of 10 μM zVAD-*fmk* (Enzo Life Sciences, Lörrach, Germany).

### 2.3. Biochemical Serum Analyses

Alanine aminotransferase (ALT), alkaline phosphatase (ALP), total bilirubin (BIL-T), cholesterol, triglycerides and glucose were measured in blood serum from non-fasted mice using standard assays in a Cobas C111 biochemical analyzer (Roche, Mannheim, Germany).

### 2.4. Antibodies

The antibodies used for immunoblotting and immunostaining procedures, together with their supplier and product number, are listed in the Table 1 below.

### 2.5. Immunoblotting

Protein extracts from primary hepatocytes or liver tissues were prepared in a standard lysis buffer containing 20 mM HEPES-KOH pH 7.6, 150 mM NaCl, 1.5 mM MgCl_2_, 1 mM EGTA, 1% Triton X-100, 10% glycerol, phosSTOP phosphatase inhibitors (Roche, Basel, Switzerland) and complete protease inhibitors (Roche). Lysates were separated by SDS-polyacrylamide gel electrophoresis (PAGE), transferred to Immobilon-P PVDF membranes (Millipore, Burlington, MA, USA), and analyzed by immunoblotting. Membranes were probed with primary antibodies against the following proteins: NEMO, p62, phospho-p62 (S351), S 6, phospho-S6, AMPK, phospho-AMPK, IKK2, LC3B, ATG16L1, α-Tubulin, actin and GAPDH, which were obtained from the suppliers mentioned in the antibody table. Membranes were then incubated either with secondary HRP-coupled antibodies (GE Healthcare, Chicago, IL, USA and Jackson ImmunoResearch, West Grove, PA, USA) and developed with chemiluminescent detection substrate (GE Healthcare) or with secondary antibodies coupled to IRDye680 or 800 and visualized with Odyssey infrared imaging system (Licor, Lincoln, NE, USA). Protein expression in immunoblots was performed by band intensity quantification using ImageJ (Version 1.53a; NIH, Bethesda, MD, USA) [42].

### 2.6. Immunofluorescence (IF) on Liver Sections and Primary Hepatocytes

p62 IF staining was performed on 5-μm paraffin liver sections after rehydration, and antigen retrieval was performed by heating in 10 mM sodium citrate, 0.05% Tween-20 at pH 6.2, aldehyde quenching in 100 mM glycine and blocking in 1% BSA, 0.2% fish-skin gelatin, 0.2% Triton X-100 0.05% Tween-20 in PBS. Incubation with anti-p62 antibody (Progen, Heidelberg, Germany) was done overnight at 4 °C, followed by incubation with anti-guinea pig secondary antibody coupled to Alexa-594 (Molecular Probes, Eugene, OR, USA). The sections were mounted in Vectashield containing DAPI (Vector Laboratories, Burlingame, CA, USA).

LC3B IF staining was performed on primary hepatocytes cultured on coverslips and fixed in 4% paraformaldehyde. Reactive aldehydes were then quenched with 50 mM NH_4_Cl and the cells were permeabilized with 0.1% Triton-X100 and blocked in 0.2% fish-skin gelatin diluted in PBS. Incubation with anti-LC3B antibody (Nanotools, Munich, Germany) was performed for 25 min at RT, followed by incubation with anti-mouse secondary antibody coupled to Alexa-488 (Molecular Probes), and the sections were mounted in Vectashield containing DAPI.

### 2.7. Histology and Immunohistochemistry (IHC)

H & E, Masson’s trichrome and Oil Red O stainings, as well as IHC, were performed by standard procedures. Tissues were fixed overnight in 4% paraformaldehyde, embedded in paraffin, and cut into 5-μm sections. H & E-stained sections were examined in a blinded fashion for the amount of steatosis, inflammation, tissue damage, fibrosis and tumorigenesis. Collagen was visualized using a Masson’s trichrome stain kit (Sigma-Aldrich, HT15-1KT) according to the manufacturer’s instructions. Visualisation of neutral lipids was performed on 10-μm-thick frozen liver sections that were fixed in 4% paraformaldehyde for 10 min, followed by staining with 0.3% Oil Red O (Sigma-Aldrich) in isopropanol/water (60:40 vol/vol) for 15 min.

For IHC staining, paraffin sections were rehydrated and antigen retrieval was performed either by heating in 10 mM sodium citrate, 0.05% Tween-20 at pH 6.2, or by incubation with 20 µg/mL proteinase K (Roche). Antibodies against the following proteins were used: Ki-67, cleaved caspase-3, α-SMA, anti-F4/80, p62, GFP and Nqo1, which were obtained from the suppliers mentioned in the antibody table. Biotinylated secondary antibodies were purchased from Perkin Elmer, DAKO or Vector Laboratories. Stainings were visualized with the ABC Kit Vectastain Elite and DAB substrate (Vector Laboratories). For optimal visualization, levels and brightness/contrast adjustments of the IF and IHC pictures were equally applied using Adobe Photoshop.

### 2.8. Image Quantification

IHC quantification was performed on three randomly selected high-power fields (HPF) per liver sample. Liver sections from a minimum of three mice per genotype were analyzed. Quantification of cleaved caspase-3^+^ apoptotic hepatocytes was performed manually. Ki-67^+^ proliferating hepatocytes, α-SMA^+^ activated hepatic stellate cells and F4/80^+^ macrophages were quantified using measure and analyze particles functions in ImageJ software. Ki-67 quantification is presented as number of hepatocyte positive nuclei per high power field, whereas α-SMA and F4/80 data are presented as % of covered area for each cell type over total tissue area.

Quantification of autophagosome number in primary hepatocytes was performed on three randomly selected viewing fields per condition, capturing at least 30 cells. Quantification was done using ImageJ software by applying the appropriate pixel threshold equally on all selected pictures and using the analyze particle function; 3–5 independent experiments were quantified.

### 2.9. Quantification of Macroscopically Visible Tumors and Histopathological Evaluation

Livers of 40–52-week-old mice were excised, digitally photographed with their dorsal side exposed, and weighed to calculate the liver/body weight ratio. The tumor number and size were determined by counting the number of visible tumors/nodules and measuring the diameter of the largest tumor using ImageJ. Liver tumors were clustered in four size groups based on their diameter (small: <2 mm, medium: 2–5 mm, big: 5–10 mm, very big: >10 mm). Histopathological evaluation was performed as previously described [8].

### 2.10. Electron Microscopy

For electron microscopy, 3-mm-long liver samples from the left lobe were excised from NEMO^FL^ and NEMO^LPC-KO^ mice and fixed in 2.5% glutaraldehyde/2% paraformaldehyde in phosphate buffer (pH 7.4) for 3 h at room temperature. The tissues were post-fixed in 1% OsO_4_ and embedded in Epon resin. Then, 70–90-nm sections were cut, stained with uranyl acetate and lead citrate and viewed in the CECAD Imaging facility under a JEM-2100Plus transmission electron microscope (Jeol, Tokyo, Japan). Representative pictures were captured using a OneView camera (Gatan Inc., Pleasanton, CA, USA).

### 2.11. Quantitative RT–PCR

Total RNA was extracted from primary hepatocytes using NucleoSpin^®^ RNA II Total RNA isolation Kit (Macherey-Nagel) following the manufacturer’s protocol, and cDNA was prepared with Superscript III cDNA-synthesis Kit (Invitrogen, Waltham, MA, USA). All real-time PCR reactions were performed using SYBR Green PCR Master Mix (Applied Biosystems, Waltham, MA, USA) and the following gene-specific primers: *Nqo1* for: AGCGTTCGGTATTACGATCC, *Nqo1* rev: AGTACAATCAGGGCTCTTCTCG, *Pgd* for: AAAGATCCGGGACAGTGCT, *Pgd* rev: CACCGAGCAAAGACAGCTT, *Gclc* for: GTGGACGAGTGCAGCAAG, *Gclc* rev: GTCCAGGAAATACCCCTTCC, *Gstm1* for: CACAAGATCACCCAGAGCAA, *Gstm1* rev: TGGTTCTCCACAATGTCTGC, *Sqstm1* for: GCTGCCCTATACCCACATCT, *Sqstm1* rev: CGCCTTCATCCGAGAAAC, *Afp* for: CTCAGCGAGGAGAAATGGTC, *Afp* rev: GAGTTCACAGGGCTTGCTTC, *Gpc3* for: CTGAGCCGGTGGTTAGCC, *Gpc3* rev: TCACTTTCACCATCCCGTCA. Peptidylprolyl isomerase A (*Ppia*) was used as a reference gene (*Ppia* for: ATGGTCAACCCCACCGTGT, *Ppia* rev: TTTCTGCTGTCTTTGGAACTT). Relative expression of gene transcripts was assessed by using the 2^−ΔΔCT^ method.

### 2.12. Statistical Analysis

For comparisons between two datasets, unpaired Student’s t-test or Mann–Whitney U test was used depending on whether the datasets fulfilled the D’Agostino–Pearson normality test criteria or not. For comparisons of multiple datasets that followed Gaussian distribution or not, one-way ANOVA with a post-hoc Tukey’s test or a Kruskal–Wallis test with a post-hoc Dunn’s test was performed, respectively. In all dot plots, horizontal lines represent the mean values, while column graphs represent the mean ± standard error of the mean (SEM). A *p* value of less than 0.05 was considered significant (* *p* ≤ 0.05, ** *p* ≤ 0.01, *** *p* ≤ 0.005). Statistical analysis was performed with Prism version 6.0 (GraphPad, San Diego, CA, USA).

## 3. Results

### 3.1. Focal p62 Accumulation and mTOR Activation in Hepatocytes of NEMO^LPC-KO^ Mice without Autophagy Inhibition

**Figure 1:**
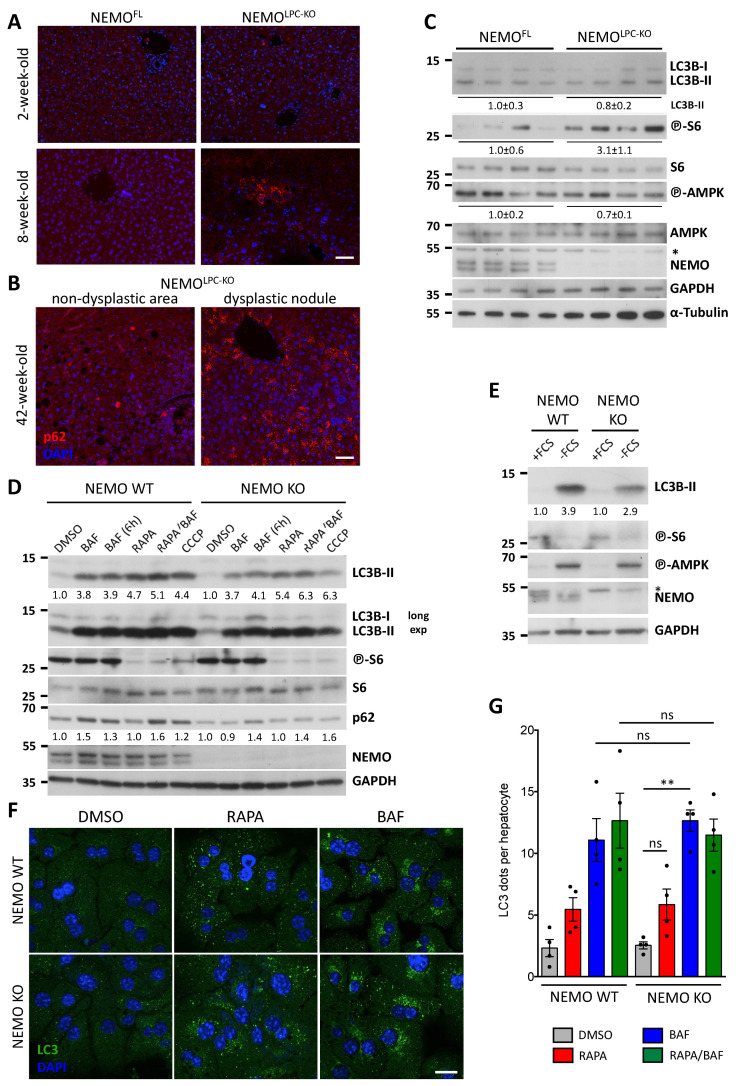
Progressive accumulation of p62, but no impairment in autophagosome formation in hepatocytes of NEMO^LPC-KO^ mice. (**A**,**B**) Representative images of IF immunostaining for p62 performed on liver sections from 2-, 8- and 42-week-old NEMO^LPC-KO^ mice and age-matched controls. Note in (**B**) the strong increase in p62 aggregates in hepatocytes of tumor/dysplastic areas compared to non-tumor areas of NEMO^LPC-KO^ mice. DAPI is used to stain the nuclei. (**C**–**E**) Immunoblot analysis of the indicated proteins in total liver lysates from 8-week-old NEMO^LPC-KO^ mice and age-matched controls (**C**), or in total lysates from control (WT) and NEMO-deficient (KO) primary hepatocytes treated with bafilomycin A1 (BAF) for 2 or 6 h, rapamycin (RAPA) for 2 h, CCCP for 2 h (**D**), and upon serum (FCS) starvation for 24 h (**E**). Asterisks indicate non-specific bands. GAPDH and α-tubulin were used as loading controls. Representative results from three independent experiments are shown in (**D**,**E**). In (**C**), band quantification represents the mean intensity ± standard error of the mean (SEM) per genotype relative to control mice and normalized to GAPDH. In (**D**,**E**), band quantification represents intensity normalized to GAPDH and relative to DMSO- or +FCS-treated sample for each genotype. (**F**) Representative pictures of IF immunostaining for LC3 in WT and NEMO KO hepatocytes upon treatment with BAF or RAPA for 2 h. DAPI is used to stain the nuclei. (**G**) Quantification of autophagosomes (LC3 dots) in experiments described in F. Graphs depict mean ± SEM (*n* = 4). Each dot represents the average value per experimental condition in one experiment. ns, not significant; ** *p* ≤ 0.01. Uncropped scans of the immunoblots are shown in Figure S5. Bars: 50 μm (**A**,**B**); 20 μm (**F**).

Aberrant p62 expression and mTOR activation have been shown to drive hepatocarcinogenesis [43,44]. To assess whether p62 accumulation is associated with the CLD pathology and tumorigenesis in NEMO^LPC-KO^ mice, we examined its expression in livers of 2-, 8- and 42-week-old mice. Small foci of p62-positive hepatocytes were observed in hepatocytes from 8 weeks old, but not from 2-week-old NEMO^LPC-KO^ mice (Figure 1A). In 42-week-old NEMO^LPC-KO^ mice, some dysplastic nodules exhibited hepatocytes with a much higher number of cytoplasmic p62 aggregates compared to neighboring non-dysplastic areas (Figure 1B). Moreover, higher levels of phosphorylated S6 were detected in livers from 8-week-old NEMO^LPC-KO^ mice compared to their littermate controls, indicative of stronger mTORC1 activation. In contrast, no consistent differences in the autophagosome marker LC3B-II and in AMPK activation were observed between NEMO^LPC-KO^ and control mice (Figure 1C).

Because NEMO has been implicated in autophagosome formation [9], we examined the competence of NEMO-deficient primary hepatocytes to form autophagosomes upon treatment with the mTOR inhibitor rapamycin, the inhibitor of autophagosome–lysosome fusion bafilomycin A1 (BafA1), the mitochondrial uncoupler and mitophagy inducer carbonyl cyanide *m*-chlorophenyl hydrazone (CCCP), and upon serum starvation (FCS withdrawal). Interestingly, no significant difference was observed in the induction of LC3B-II under all these conditions in NEMO-deficient compared to wild-type (WT) hepatocytes. Moreover, mTOR inhibition by rapamycin or CCCP and AMPK activation upon serum starvation were not affected by NEMO deficiency (Figure 1D,E). A comparable induction of autophagosomes, as assessed by the quantification of LC3 fluorescent puncta, was also seen upon rapamycin and BafA1 treatment of WT and NEMO-deficient hepatocytes (Figure 1F,G).

Autophagy is also involved in triglyceride mobilization from lipid droplets (LDs) through a process called lipophagy, and autophagy impairment was shown to exacerbate starvation-induced LD accumulation in hepatocytes [13,45]. Therefore, we fasted WT and NEMO^LPC-KO^ mice and performed Oil Red O staining for neutral lipids on liver sections. However, no increase in LD content was observed in NEMO-deficient hepatocytes, arguing against a lipophagy defect (Figure S1A). Accordingly, we could identify a similar number of typical autophagosomes and lipophagy profiles upon electron microscopy analysis of liver biopsies from fasted NEMO^LPC-KO^ mice and littermate controls (Figure S1B). Moreover, starvation-induced autophagy led to efficient clearance of p62 aggregates that were present in hepatic foci of 8-week-old NEMO^LPC-KO^ mice (Figure S1C).

In addition, we examined the contribution of IKK2 or IKK1 to rapamycin- and BafA1-induced autophagosome formation. Similar to NEMO deficiency, no significant differences were observed in LC3B-II levels and in the number of autophagosomes in IKK2- (Figure S2A–C) or IKK1-deficient hepatocytes or in hepatocytes expressing constitutively active IKK2 (IKK2ca; Figure S2D,E). Of note, total p62 levels appeared to be lower both in NEMO- and IKK2-deficient hepatocytes compared to controls (Figure 1D and Figure S2A), likely due to NF-κB-dependent regulation of *p62* gene transcription [46]. Overall, these results suggest that autophagosome formation in hepatocytes is not considerably affected by the lack of IKK subunits.

### 3.2. Hepatic NEMO and Autophagy Protect from CLD via Distinct Mechanisms

**Figure 2:**
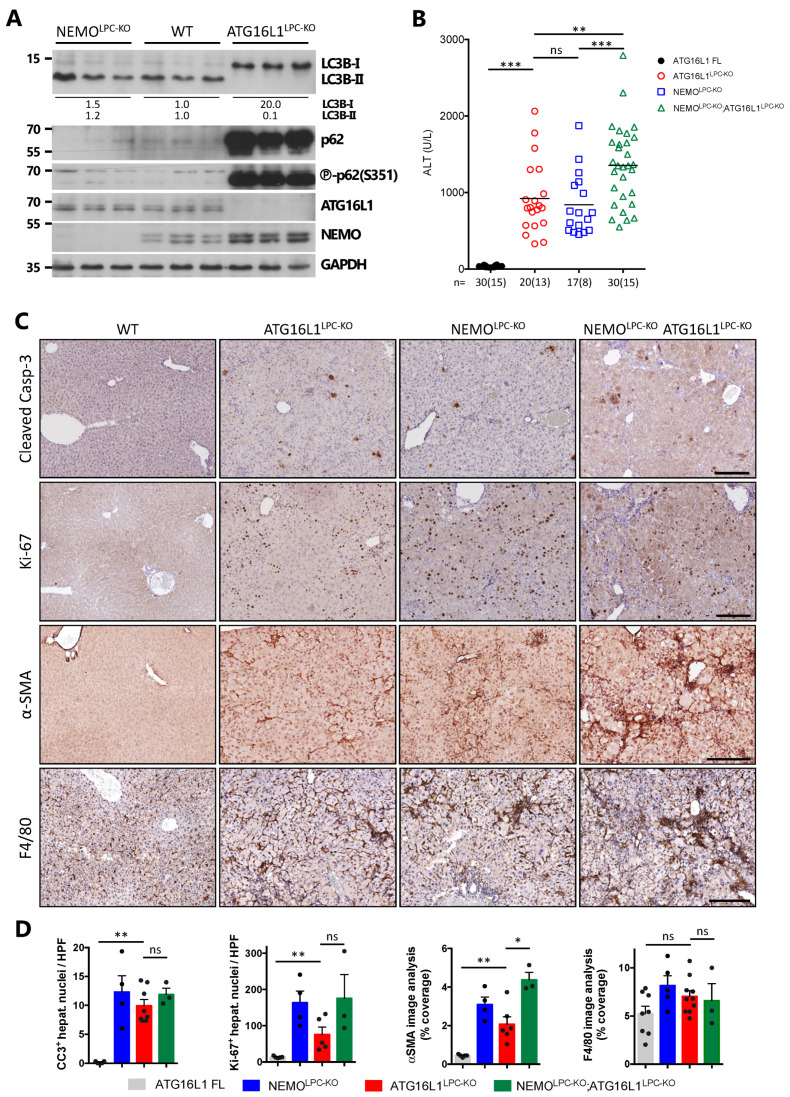
NEMO deficiency and autophagy inhibition independently lead to spontaneous liver damage, hepatitis and fibrosis. (**A**) Immunoblot analysis of the indicated proteins in total liver lysates from 8-week-old WT, NEMO^LPC-KO^ and ATG16L1^LPC-KO^ mice. GAPDH is used as loading control. Band quantification represents the mean intensity per genotype relative to control mice and normalized to GAPDH. Uncropped scans of the immunoblots are shown in Figure S5. (**B**) Serum alanine aminotransferase (ALT) levels in 8-week-old mice with the indicated genotypes. Each symbol represents one mouse and horizontal lines indicate mean values. *n* indicates the number of analyzed mice, out of which the number of males is shown in brackets. (**C**) Representative images of liver sections from 8-week-old mice with the indicated genotypes after immunostaining with the indicated antibodies. (**D**) Quantification of the depicted markers in (**C**). Graphs show mean ± SEM (*n* = 3–8 mice per genotype). Each dot represents the average value per analyzed mouse with the indicated genotype. ns, not significant; * *p* ≤ 0.05, ** *p* ≤ 0.01, *** *p* ≤ 0.005. Bars: 200 μm.

As our results were at odds with the proposed contribution of IKK subunits in autophagosome formation, we decided to compare side-by-side the phenotype of NEMO^LPC-KO^ mice with mice lacking an essential autophagy gene in LPCs. To this end, we generated ATG16L1^LPC-KO^ mice, which lack ATG16L1 in hepatocytes and cholangiocytes. As expected, strong inhibition of LC3B-I lipidation to LC3B-II and massive accumulation of p62 were detected in liver lysates of 8-week-old ATG16L1^LPC-KO^ mice (Figure 2A). p62 was also heavily phosphorylated at S351 (S349 in human), which increases its binding to Keap1 leading to Nrf2 overactivation [26,47]. In contrast, NEMO^LPC-KO^ mice exhibited predominantly the LC3B-II form similar to WT mice. In line with the notion that the IKK subunit levels are regulated by autophagy [11], NEMO protein levels were higher in ATG16L1-deficient livers compared to controls (Figure 2A).

As previously described in ATG7^Δhepa^ mice [18,19], ATG16L1^LPC-KO^ mice developed spontaneous liver damage, which was comparable to NEMO^LPC-KO^ mice, based on serum ALT levels and the number of cleaved caspase-3 (CC3)^+^ hepatocytes (Figure 2B–D). Hepatocyte death in ATG16L1^LPC-KO^ mice was accompanied by an increase in Ki-67^+^ proliferating hepatocytes, albeit lower than in NEMO^LPC-KO^ mice. An increase in fibrosis-associated, activated hepatic stellate cells (HSC) and a focal accumulation of macrophages was also observed, as indicated by immunostaining for α-smooth muscle actin (α-SMA) and F4/80, respectively (Figure 2C,D).

**Figure 3:**
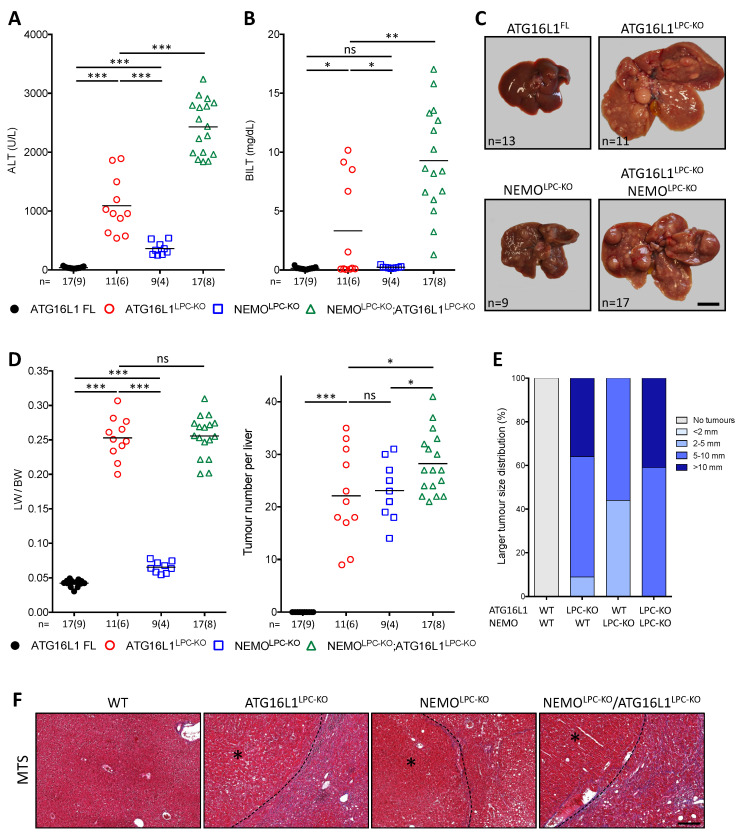
Combined NEMO deficiency and autophagy inhibition leads to aggravated CLD and hepatocarcinogenesis. (**A**,**B**) Serum ALT (**A**) and total bilirubin (**B**) levels in 40–52-week-old mice with the indicated genotypes. Each symbol represents one mouse and horizontal lines indicate mean values. (**C**) Representative liver images from 52-week-old mice with the indicated genotypes. (**D**,**E**) Tumor load in mice with the indicated phenotypes as estimated by quantification of the LW/BW ratio and tumor number per liver (**D**), and the larger tumor size distribution (**E**). Each symbol represents one mouse and horizontal lines indicate mean values. (**F**) Representative images of Masson’s trichrome stained (MTS) liver sections from 40–52-week-old mice with the indicated genotypes. HCC/dysplastic nodule areas are marked with an asterisk. In (**A**,**B**,**D**), *n* indicates the number of analyzed mice, out of which the number of males is shown in brackets. ns, not significant; * *p* ≤ 0.05, ** *p* ≤ 0.01, *** *p* ≤ 0.005. Bars: (**C**) 1 cm; (**F**) 200 μm.

Next, we looked at hepatocarcinogenesis in aged ATG16L1^LPC-KO^ mice. In contrast to 1-year-old NEMO^LPC-KO^ mice that show reduced serum ALT levels compared to 8-week-old mice, liver injury in ATG16L1^LPC-KO^ mice remained high throughout ageing (Figure 3A). Additionally, 4 out of 11 mice developed high bilirubinemia and jaundice, which was not observed in NEMO^LPC-KO^ mice (Figure 3B). This condition appeared rather late during CLD development in ATG16L1^LPC-KO^ mice, as mice up to 40 weeks of age did not appear icteric. Moreover, 40–52-week-old ATG16L1^LPC-KO^.

Mice presented liver tumors comparable in number but slightly larger in size to those of NEMO^LPC-KO^ mice of the same age. However, unlike the latter, ATG16L1^LPC-KO^ mice showed severe hepatomegaly, which was reflected in the very high liver-to-body weight (LW/BW) ratio (Figure 3C–F). Histopathological evaluation revealed the presence of multiple regenerative nodules, ductular proliferations, fibrosis and ceroid-laden macrophages in the livers of ATG16L1^LPC-KO^ mice. Although liver tumors in ATG7^Δhepa^ mice have been described as benign adenomas [18,19,20], we identified at least one well-differentiated HCC in 40% of ATG16L1^LPC-KO^ mice (Figure 3F).

To address whether autophagy inhibition and NEMO deletion act synergistically in the development of spontaneous liver disease, we generated mice lacking both ATG16L1 and NEMO in LPCs (ATG16L1^LPC-KO^ NEMO^LPC-KO^) and compared them to the respective single knockouts. Strikingly, we found an aggravation in several aspects of the liver pathology in ATG16L1^LPC-KO^ NEMO^LPC-KO^ mice. In 8-week-old mice, liver injury and fibrosis were significantly increased (Figure 2B–D), while aged mice had higher hepatocellular damage and all of them developed high bilirubinemia even before the age of 40 weeks (Figure 3A,B). The number of liver tumors was also significantly increased, although the tumor size, hepatomegaly and LW/BW ratio were comparable to ATG16L1^LPC-KO^ mice (Figure 3C–E). These results are consistent with a synergistic interaction between autophagy and the NEMO-regulated pathway to independently protect against liver cell damage, thereby preventing CLD development.

### 3.3. p62 Deficiency Impairs Survival of NEMO^LPC-KO^ Mice

Ablation of p62 was shown to have a beneficial effect on the CLD pathology of ATG7^Δhepa^ mice by significantly attenuating liver damage, fibrosis and hepatomegaly, and liver tumor progression [18,21]. To examine if p62 deficiency would have a similar effect in NEMO^LPC-KO^ mice, which do not exhibit an underlying autophagy inhibition, we generated NEMO^LPC-KO^ p62^KO^ mice. Unexpectedly, these mice showed increased perinatal lethality with only a small number of them reaching post-weaning age, thereby preventing us from drawing firm conclusions (Figure S3). Of note, the single survivor that reached the age of 1 year presented with very big liver tumors (Figure S3B). These data suggest that in contrast to its detrimental effect in CLD mouse models with an underlying autophagy impairment, p62 plays a protective role against CLD progression in NEMO^LPC-KO^ mice, and systemic p62 deficiency is synthetically lethal when combined with LPC-specific NEMO deficiency.

### 3.4. Expression of a p62 Mutant Aggravates Hepatocarcinogenesis in NEMO^LPC-KO^ Mice

**Figure 4:**
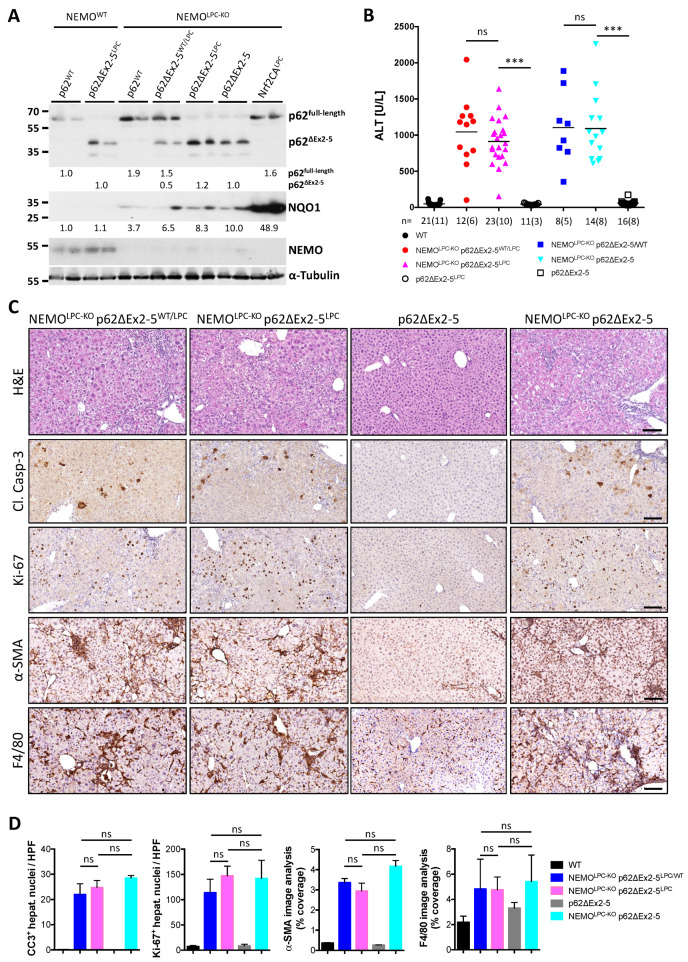
Expression of the p62ΔEx2-5 mutant does not prevent the liver pathology in NEMO^LPC-KO^ mice. (**A**) Immunoblot analysis of p62, NQO1 and NEMO in total liver lysates from 8-week-old mice with the indicated genotypes. α-Tubulin is used as the loading control. Band quantification represents the mean intensity per genotype relative to control mice and normalized to α-tubulin. Uncropped scans of the immunoblots are shown in Figure S5. (**B**) Serum ALT levels in 8-week-old mice with the indicated genotypes. Each symbol represents one mouse and horizontal lines indicate mean values. *n* indicates the number of analyzed mice, out of which the number of males is shown in brackets. (**C**) Representative images of liver sections from 8-week-old mice with the indicated genotypes after immunostaining with the indicated antibodies. (**D**) Quantification of the depicted markers in (**C**). Graphs show mean ± SEM (*n* = 3–8 mice per genotype). Each dot represents the average value per analyzed mouse with the indicated genotype. ns, not significant; *** *p* ≤ 0.005. Bars: 200 μm.

p62 is a multifunctional protein, and several molecular interactions have been described to regulate signaling pathways that affect cell survival (Figure S4A). To address the in vivo relevance of selected p62 functions, we used mice bearing *LoxP* sites before exon 2 and after exon 5 in the *Sqstm1/p62* locus. Removal of exons 2–5 upon Cre-mediated recombination leads to stable expression of a truncated p62 mutant (p62ΔEx2-5) (Figure 4A), which lacks the portion of the protein that corresponds to the end of the PB1 domain, the first nuclear localization signal (NLS), and the interacting domains for Raptor, RIPK1 and TRAF6 (Figure S4A, region between D69 and L251). p62ΔEx2-5 mutant protein is expressed at similar levels to full-length p62 protein in the liver (Figure 4A). To assess the effect of p62ΔEx2-5 expression, we first generated mice expressing p62ΔEx2-5 in all cells (p62^ΔEx2-5^) using Cre *Deleter* and compared them to whole-body p62-deficient (p62^KO^) mice. p62^KO^ mice develop a late-onset obesity and insulin resistance phenotype that is normally prevented through a p62/ERK inhibitory interaction [36]. Heterozygous mice were used as controls. As described before, both male and female p62^KO^ mice developed obesity characterized by a significant increase in body weight, which was particularly striking after 5 months of age (Figure S4B), steatosis and increased liver size without affecting the LW/BW ratio (Figure S4C). Significantly higher total cholesterol levels were observed in both male and female p62^KO^ mice. However, no statistically significant differences in triglyceride and non-fasting glucose levels were observed, although a trend for increased levels was seen in male p62^KO^ mice (Figure S4D). In contrast, the average body weight of male p62^ΔEx2-5^ mice was between p62^KO^ and control mice, and statistically deviated from p62^KO^ much earlier than from control males. p62^ΔEx2^^-^^5^ female mice were significantly leaner than p62^KO^ females, and were comparable to control females (Figure S4B). Furthermore, most p62^ΔEx2-5^ mice had a similar liver weight to sex-matched control mice and lower compared to p62^KO^ mice, while no significant difference was observed in total cholesterol, triglycerides, glucose and liver injury markers (Figure S4C,D). Altogether, these data show that p62^ΔEx2-5^ mice do not develop obesity and fatty liver as p62^KO^ mice do, supporting the dependence of this phenotype on p62/ERK inhibitory interaction [36], which is expected to remain unaffected in the p62ΔEx2-5 mutant.

To disentangle the effect of the different p62-regulated pathways and to circumvent the premature lethality of ubiquitous p62 ablation in the context of NEMO-dependent CLD, we generated NEMO^LPC-KO^ mice with LPC-specific or whole-body expression of p62ΔEx2-5 (NEMO^LPC-KO^ p62ΔEx2-5^LPC^ and NEMO^LPC-KO^ p62ΔEx2-5, respectively; Figure 4A). Unlike the early lethality observed in NEMO^LPC-KO^ p62^KO^ mice, no deviation from the expected Mendelian birth ratio or perinatal lethality was observed in these strains. This suggests that a p62 function that is maintained in the p62ΔEx2-5 mutant is sufficient to prevent lethality in NEMO^LPC-KO^ mice. Nevertheless, determination of serum ALT levels (Figure 4B) and liver immunohistochemical analyses in livers of 8-week-old mice revealed that systemic or LPC-specific expression of p62ΔEx2-5 did not significantly alter liver injury or other CLD indicators, such as compensatory proliferation, inflammation and fibrosis, in NEMO^LPC-KO^ mice (Figure 4C,D).

**Figure 5:**
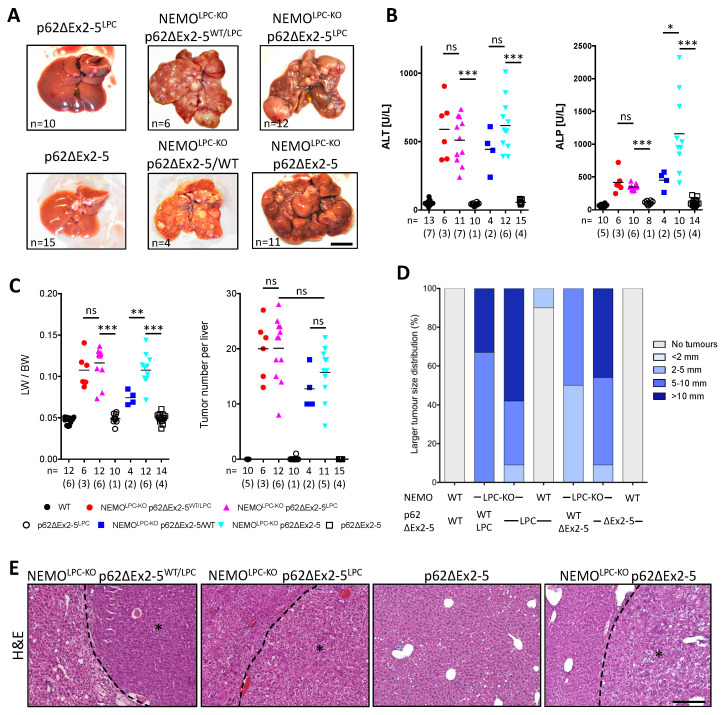
p62ΔEx2-5 expression aggravates liver disease and hepatocarcinogenesis in aged NEMO^LPC-KO^ mice. (**A**) Representative liver images from 1-year-old mice with the indicated genotypes. (**B**–**D**) Serum ALT and alkaline phosphatase (ALP) levels (**B**), LW/BW ratio and tumor number per liver (**C**), and larger tumor size distribution (**D**) in 1-year-old mice with the indicated genotypes. Each symbol represents one mouse and horizontal lines indicate mean values. *n* indicates the number of analyzed mice, out of which the number of males is shown in brackets. (**E**) Representative images of H & E-stained liver sections from 1-year-old mice with the indicated genotypes. HCC areas are marked with an asterisk. ns, not significant; * *p* ≤ 0.05, ** *p* ≤ 0.01, *** *p* ≤ 0.005. Bars: (**A**) 1 cm; (**E**) 200 μm.

Hepatocarcinogenesis was subsequently evaluated in 1-year-old NEMO^LPC-KO^ p62ΔEx2-5^LPC^ and NEMO^LPC-KO^ p62ΔEx2-5 mice (Figure 5). Strikingly, p62ΔEx2-5 expression enhanced carcinogenesis, leading to the development of significantly more large-sized liver tumors in both NEMO^LPC-KO^ strains (compare Figure 3C and Figure 5A). This was evident in the increased LW/BW ratio and the fact that about 50% of these mice developed tumors with >10-mm diameter (Figure 5C,D), which was barely seen in NEMO^LPC-KO^ mice. In contrast, the tumor number was not significantly altered by the p62ΔEx2-5 expression, implying that this p62 mutant promotes liver tumor progression rather than the tumor initiation (Figure 5C). Histopathological evaluation confirmed that these large tumors were HCCs (Figure 5E). Interestingly, the liver injury observed in aged NEMO^LPC-KO^ mice was also aggravated in particular by the systemic p62ΔEx2-5 expression. More specifically, in addition to high ALT levels, seven out of nine NEMO^LPC-KO^ p62ΔEx2-5 mice exhibited very high alkaline phosphatase (ALP) levels (Figure 5B), although only one of them presented with high total bilirubin (>1.2 mg/dL).

Overall, these data show that expression of the p62ΔEx2-5 mutant aggravates hepatocarcinogenesis, while it additionally promotes the development of anicteric cholestatic liver disease in NEMO^LPC-KO^ mice, mainly by acting in non-LPC compartment(s). The combination of cholestasis and hepatocellular damage at advanced stages of CLD could fuel the development of more aggressive tumors, as previously shown in TAK1^LPC-KO^ mice [48].

### 3.5. Nrf2 Overactivation Per Se Does Not Affect Liver Damage and Tumorigenesis in NEMO^LPC-KO^ Mice

**Figure 6:**
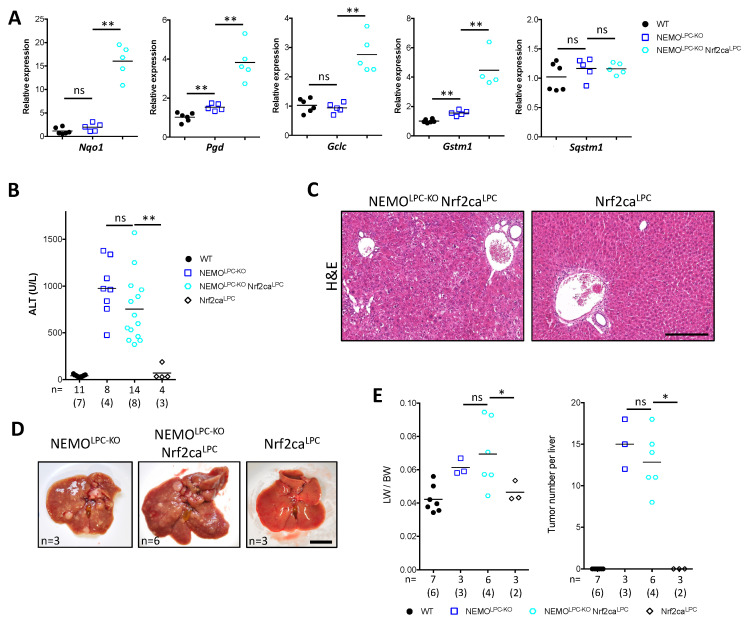
LPC-specific Nrf2ca expression does not alter liver damage and hepatocarcinogenesis in NEMO^LPC-KO^ mice. (**A**) Expression of *Sqstm1* and Nrf2 target genes quantified by qRT-PCR on liver samples with the indicated genotypes. Relative expression normalized to *Ppia* expression is presented and compared to age-matched WT control samples (*n* = 5–6). (**B**) Serum ALT levels in 8-week-old mice with the indicated genotypes. Each symbol represents one mouse and horizontal lines indicate mean values (*n* = 4–19 mice). (**C**) Representative images of H & E-stained liver sections from 8-week-old mice with the indicated genotypes. (**D**,**E**) Representative liver images (**D**), LW/BW ratio and tumor number per liver (**E**) in 1-year-old mice with the indicated genotypes. In B and E, *n* indicates the number of analyzed mice, out of which the number of males is shown in brackets. ns, not significant; * *p* ≤ 0.05, ** *p* ≤ 0.01. Bars: 200 μm (**C**), 1 cm (**D**).

Generation of reactive oxygen species (ROS) has been implicated in liver damage observed in NEMO^LPC-KO^ mice, as the feeding of these mice with the antioxidant food preservative butylate hydroxyanisole (BHA) conferred protection from hepatocyte apoptosis [5]. BHA is also a known Nrf2 activator [49]. To examine the activation status of the Nrf2 pathway in NEMO^LPC-KO^ mice, we looked at the expression of a panel of antioxidant response and Nrf2 target genes (*Nqo1*, *Pgd*, *Gclc*, *Gstm1*) that are shown to be strongly upregulated in livers of ATG7^Δhepa^ mice [26,47]. Unlike ATG7^Δhepa^ mice, the mRNA expression of Nrf2 target genes and *Sqstm1/p62* in livers of 8-week-old NEMO^LPC-KO^ mice was not significantly increased (Figure 6A). This result was confirmed by analysis of NQO1 protein levels, which were only mildly elevated in liver lysates of NEMO^LPC-KO^ mice as compared to controls (Figure 4A).

In ATG5^Δhepa^ or ATG7^Δhepa^ mice, Nrf2 overactivation has been shown to promote both liver injury and hepatocarcinogenesis [20,26,50]. To test whether forced Nrf2 overactivation alone could promote liver damage in a CLD model without underlying autophagy inhibition, we generated NEMO^LPC-KO^ Nrf2ca^LPC^ mice that express a constitutively active Nrf2 mutant in LPCs [51]. While Nrf2ca expression led to a robust upregulation of Nrf2 target gene expression (Figure 4A and Figure 6A), it neither aggravated nor reduced the liver damage in 8-week-old NEMO^LPC-KO^ mice (Figure 6B,C). Similarly, no significant exacerbation was observed in liver tumorigenesis in 1-year-old NEMO^LPC-KO^ Nrf2ca^LPC^ mice (Figure 6D,E). Notably, despite the strongly increased expression of classical Nrf2 target genes, Nrf2 overactivation in NEMO^LPC-KO^ Nrf2ca^LPC^ mice did not lead to increased expression of p62 both at the mRNA and protein level, as compared to NEMO ^LPC-KO^ mice (Figure 4A and Figure 6A). Unlike previously suggested [52], this argues against p62 being a direct Nrf2 target gene, at least in primary hepatocytes. Alternatively, as p62 expression appears to be regulated by IKK/NF-κB signaling [46], our result may reflect an epistatic effect of NF-κB-mediated p62 transcription compared to Nrf2 regulation in Nrf2ca-expressing NEMO-deficient hepatocytes. Altogether, these data suggest that Nrf2 overactivation per se does not drive liver injury and hepatocarcinogenesis in mice with autophagy-competent hepatocytes, and other molecular pathways that are deregulated in autophagy-defective hepatocytes may potentiate such effects.

### 3.6. Aberrant p62 Accumulation and Acquired Autophagy Defects Associate with p62-Keap1-Nrf2 Positive Liver Tumors

**Figure 7:**
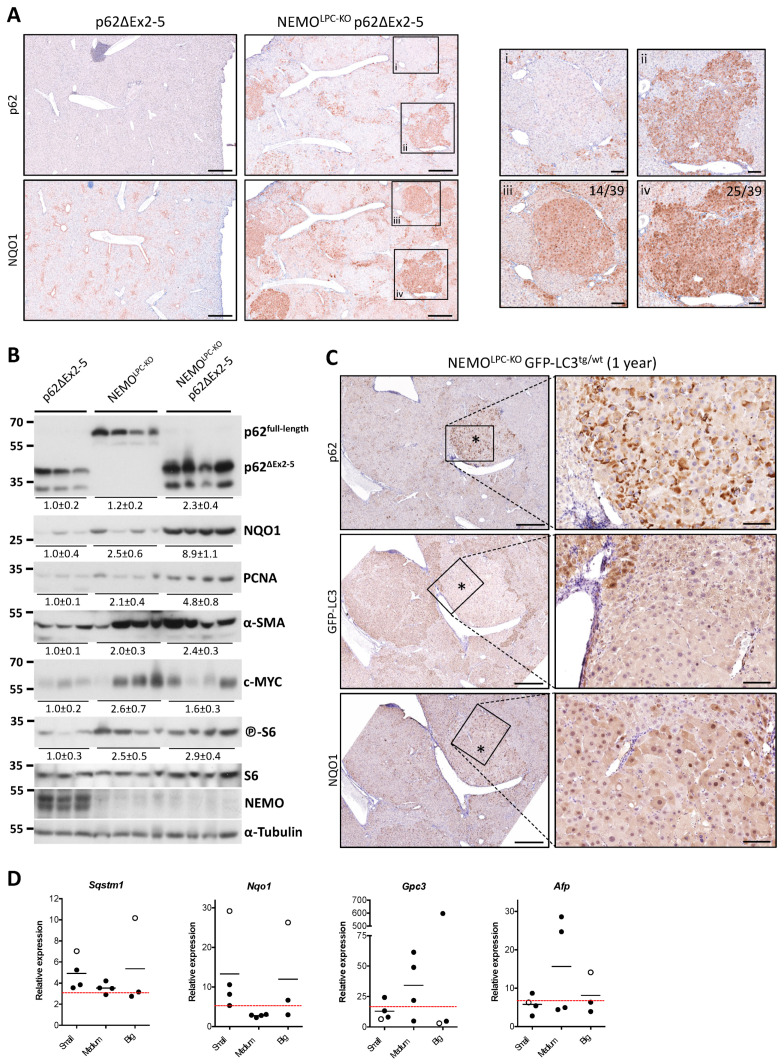
Frequent activation of the p62-Keap1-Nrf2 axis in liver tumors of NEMO ^LPC-KO^ mice correlates with an underlying autophagy impairment and low *Gpc3* expression. (**A**) Representative images of immunostaining for p62ΔEx2-5 and NQO1 performed by IHC on sequential liver sections from 1-year-old p62ΔEx2-5 or NEMO^LPC-KO^ p62ΔEx2-5 mice. Insets illustrate examples of p62^neg^ Nrf2^pos^ (i and iii) and p62^pos^ Nrf2^pos^ (ii and iv) tumors. The frequency of the two types of Nrf2^pos^ tumors is presented (*n* = 39). (**B**) Immunoblot analysis of the indicated proteins in total liver lysates from 1-year-old mice with the indicated genotypes. α-Tubulin is used as the loading control. Band quantification represents the mean intensity ± SEM per genotype relative to control mice (p62ΔEx2-5) and normalized to α-tubulin. Uncropped scans of the immunoblots are shown in Figure S5. (**C**) Representative images of immunostaining for p62, GFP-LC3 and NQO1 performed by IHC on sequential liver sections from 1-year-old NEMO^LPC-KO^ GFP-LC3^tg/wt^ mice. Note that the dysplastic nodule (asterisk) with high accumulation of p62 aggregates and increased NQO1 expression is negative for GFP-LC3, indicative of an underlying autophagy impairment. (**D**) qRT-PCR analysis of the expression of *Sqstm1*, *Nqo1* and the liver tumor markers *Gpc3* and *Afp* in 11 randomly selected tumors of various sizes (small, <2-mm; medium, 2–5-mm; big, >5-mm) excised from 1-year-old NEMO^LPC-KO^ mice (*n* = 3). Ct values are normalized to *Ppia* and presented as relative expression compared to the expression of each gene in control liver that is set as 1. The red dashed lines indicate the average expression of each gene in non-tumor areas of the same NEMO^LPC-KO^ livers. Each circle represents the value in one mouse and the open circles denote the samples with high *Sqstm1* and *Nqo1* mRNA expression. ns, not significant; * *p* ≤ 0.05. Bars: (**A**,**C**) 500 μm; (insets) 100 μm.

The p62-Keap1-Nrf2 axis has been intrinsically linked to hepatocarcinogenesis in mouse models that exhibit some degree of autophagy impairment, but it is yet unclear if it can promote HCC in autophagy-competent livers [18,21,44,53]. While expression of Nrf2ca alone did not aggravate tumorigenesis in NEMO^LPC-KO^ mice (Figure 6), p62 transduction and overexpression in hepatocytes of WT mice did lead to Nrf2-dependent spontaneous liver tumors [44]. To further explore this issue, we first examined how frequently the p62-Keap1-Nrf2 axis is activated in liver tumors of one-year-old NEMO^LPC-KO^ p62ΔEx2-5 mice, which showed higher HCC burden, by performing IHC analysis of p62 and NQO1 expression (Figure 6A). As expected, in control p62ΔEx2-5 mice, no p62 accumulation was detected, whereas NQO1 was distributed in a zonation pattern with the highest expression around the central veins. Strikingly, NQO1 expression was significantly increased and showed a broader, more diffuse pattern in the liver parenchyma of NEMO^LPC-KO^ p62ΔEx2-5 mice with many of the dysplastic lesions being highly positive. Out of 39 randomly-selected (pre)neoplastic tumor lesions with middle/high expression of NQO1 that were analyzed for p62 expression on consecutive sections, 64% consisted mostly of hepatocytes exhibiting high numbers of p62 aggregates. The remaining 36% of liver tumors were devoid of or showed very low expression of p62 (Figure 7A). Conversely, no p62-positive tumors with low NQO1 levels were observed. These results demonstrate that the p62ΔEx2-5 mutant can still form aggregates, likely because the PB1 domain remains nearly intact, and suggest that the activation of the p62-Keap1-Nrf2 pathway is highly prevalent in NEMO^LPC-KO^ p62ΔEx2-5 mice.

To investigate whether the high frequency of p62/Nrf2^high^ tumor lesions in NEMO^LPC-KO^ p62ΔEx2-5 mice could be due to p62ΔEx2-5 mutant driving a stronger Nrf2 activation compared to full-length p62, we compared the expression of NQO1 in liver lysates of 1-year-old NEMO^LPC-KO^ and NEMO^LPC-KO^ p62ΔEx2-5 mice. Indeed, NQO1 protein levels were on average 4-fold higher in NEMO^LPC-KO^ p62ΔEx2-5 mice compared to age-matched NEMO^LPC-KO^ mice. The levels of p62ΔEx2-5 were accordingly elevated compared to full-length p62 (Figure 7B), suggesting that Nrf2 activation is a consequence of a stronger accumulation of the p62 mutant rather than a gain-of-function effect. In agreement with the increased tumor burden in NEMO^LPC-KO^ p62ΔEx2-5 mice, the protein expression of the generic cell proliferation marker PCNA was 2.5-fold higher in these mice compared to NEMO^LPC-KO^ mice (Figure 7B). In addition, we looked at the levels of c-MYC and S6 phosphorylation as a downstream target of mTORC1 activation, since both were shown to be regulated by p62 overexpression [44,54]. Unlike NQO1, no consistent difference in S6 phosphorylation or c-MYC expression was seen between NEMO^LPC-KO^ p62ΔEx2-5 and NEMO^LPC-KO^ mice (Figure 7B), suggesting that these pathways cannot explain the increased hepatocarcinogenesis in NEMO^LPC-KO^ p62ΔEx2-5 mice. Finally, because p62 deficiency in hepatic stellate cells was shown to exacerbate fibrosis-mediated hepatocarcinogenesis [55], we examined the expression of α-SMA expression. However, we could also not detect a difference that would justify the increased tumor burden in NEMO^LPC-KO^ p62ΔEx2-5 mice (Figure 7B).

Although NEMO^LPC-KO^ mice have no major autophagy deficit, the activation of the p62-Keap1-Nrf2 axis could still be the result of an acquired autophagy impairment through somatic mutations during hepatocarcinogenesis. To this end, we assessed whether p62-Keap1-Nrf2 activation correlates with a potential autophagy impairment in 9–12-month-old NEMO^LPC-KO^ mice that express wild-type p62. To monitor the autophagy status in vivo, we generated NEMO^LPC-KO^ GFP-LC3 mice that ubiquitously express GFP-LC3 [38]. In agreement with the immunoblotting data (Figure 7B), IHC analysis of p62 expression levels in dysplastic nodules and HCCs of NEMO^LPC-KO^ GFP-LC3 mice showed that a lower percentage of lesions (10% of the analyzed lesions; *n* = 20) were heavily populated with hepatocytes containing p62 aggregates. Remarkably, these p62^high^ lesions showed very low reactivity for GFP-LC3, while they had upregulated NQO1 expression (Figure 7C). Altogether, these correlative findings imply that even in a liver disease model where hepatocytes are competent for autophagy, aberrant accumulation of p62 aggregates, through an autophagy impairment and maybe other mechanisms, is likely a required early event for p62-Keap1-Nrf2-mediated hepatocarcinogenesis.

Finally, to identify a potential connection between tumors with p62-Keap1-Nrf2 activation and known HCC markers, we analyzed by qRT-PCR an independent cohort of 11 tumors of various sizes excised from 1-year-old NEMO^LPC-KO^ mice (Figure 7D). Out of 11 tumors, the two that exhibited high *p62* mRNA expression (>2-fold increase compared to the mean of p62 expression in livers of NEMO^LPC-KO^ mice) also showed very strong *Nqo1* expression, indicative of Nrf2 activation. Interestingly, analysis of the expression of two HCC diagnostic markers, namely α-fetoprotein (*Afp*) and glypican-3 (*Gpc3*) [56], showed that these two p62/Nrf2^high^ tumors expressed very low *Gpc3* levels, suggesting that p62/Nrf2 activation could define specific types of liver tumors. A large-scale analysis both in mouse and human hepatic tumors would be necessary to further address this issue.

## 4. Discussion

### 4.1. IKK Complex and Autophagy Independently Regulate Liver Homeostasis

Chronic liver injury and inflammation drive CLD progression and hepatocarcinogenesis. IKK/NF-κB signaling and autophagy are two established cellular pathways that promote cell survival, and ample genetic evidence in mouse models supports their importance for liver homeostasis [1,2,3,24,57]. A reciprocal crosstalk between these two pathways has been reported [11], but this has not been assessed in vivo during liver development. While the IKK complex subunits were previously shown to contribute to autophagosome formation [9], our data presented here do not support an involvement of NEMO, IKK1 or IKK2 in constitutive or starvation-induced autophagy, mitophagy and lipophagy. This is supported by the fact that IKK1^LPC-KO^ or IKK2^LPC-KO^ mice do not exhibit a spontaneous liver phenotype [58], unlike ATG5^Δhepa^, ATG7^Δhepa^ [18,19,20] and ATG16L1^LPC-KO^ mice (this study). Although NEMO^LPC-KO^ mice show similar liver damage as ATG16L1^LPC-KO^ mice, which could indicate a linear involvement of the two gene products in the same pro-survival pathway, their combined deletion in LPCs resulted in aggravation of liver injury and inflammation demonstrates that NEMO and autophagy maintain liver homeostasis independently. Along the same line, different molecular mechanisms seem to promote liver injury in these two models. This is exemplified by the fact that combined p62 deficiency exerts a synthetically lethal effect in NEMO^LPC-KO^ mice during the first weeks after birth, while it strongly prevented liver injury in ATG7^Δhepa^ mice [21]. These results highlight that p62 can have a harmful or protective effect depending on the etiology of the underlying liver damage. Contrary to NEMO ablation, LPC-specific knockout of the upstream kinase TAK1 was shown to induce CLD and hepatocarcinogenesis through autophagy impairment by modulating AMPK and mTORC1 activation [13,14], which were not affected in NEMO-deficient hepatocytes. An alternative molecular mechanism for the TAK1-dependent regulation of autophagy has also been proposed through an inhibitory binding of TAK1-binding proteins 2 and 3 (TAB2/3) to Beclin1, which is essential for the induction of autophagosome formation. Upon various autophagy-inducing stimuli, TAB2/3 were shown to dissociate from Beclin1 and bind TAK1, leading to downstream activation of IKK/NF-κB signaling, thus allowing Beclin1 to initiate autophagy [59]. This mechanism is compatible with our data, as it places autophagy regulation at the level of TAK1 rather than downstream of the IKK complex.

Although our results do not support a critical contribution of the IKK subunits in autophagy induction, the increased protein levels of IKK2 and NEMO in ATG16L1-deficient livers suggest that autophagy may regulate IKK complex turnover. Autophagy-mediated degradation of the IKK complex is conserved in Drosophila through the interaction of the NEMO homologue (Kenny) with Atg8/LC3, thereby controlling immune deficiency (IMD) pathway activation [60]. In some cancer cells, autophagy inhibition also leads to increased NF-κB activation and resistance to anti-cancer drugs [11,61]. This could be due to the prolonged activation of the IKK subunits or the accumulation of p62 aggregates that were shown to promote NF-κB activation through various interactions [25,62] (Figure S4A). However, there are also cancer cells where autophagy impairment dampens NF-κB signaling [63], and therefore any therapeutic intervention in the direction of modulating autophagy should take into account the cancer cell type and background mutations.

### 4.2. The Role of p62 and Nrf2 in Liver Injury Is Context-Dependent

Aberrant accumulation of p62 in autophagy-deficient hepatocytes has been implicated in driving both liver damage and hepatocarcinogenesis through Nrf2 overactivation [20,24,26]. However, p62 usually activates survival pathways that protect cells from death induction [23]. Here, we provide genetic evidence that lack of p62 dramatically reduces the overall survival of NEMO^LPC-KO^ mice, suggesting that the beneficial effect observed in ATG5^Δhepa^ or ATG7^Δhepa^ mice is specifically linked to the impact of autophagy inhibition on hepatocyte homeostasis. Strikingly, expression of the p62ΔEx2-5 mutant either systemically or in LPCs did not affect the viability of NEMO^LPC-KO^ mice, indicating that functions that are maintained in this mutant play a critical pro-survival role. Despite rescuing the overall survival deficit, the p62ΔEx2-5 mutant did not prevent the liver injury observed in young adult NEMO^LPC-KO^ mice, while in aged mice it aggravated liver damage inducing cholestasis, especially when expressed ubiquitously. Although these genetic results cannot distinguish the critical p62 function(s), they emphasize at the importance of non-LPC functions of p62 to prevent biliary damage in aged NEMO^LPC-KO^ mice. Accordingly, a recent study showed that hepatic p62 protected ATG5/Tsc1^Δhepa^ mice from liver damage and hepatocarcinogenesis [64]. Considering the detrimental impact of p62 in ATG5^Δhepa^ mice, its protective effect in these double knockout mice is likely independent of the autophagy impairment and related to the lack of Tsc1 and the ensuing constitutive activation of mTORC1.

Regarding the role of Nrf2 overactivation, our results show that activation of this transcription factor per se is not sufficient to promote liver injury. Hepatocyte-specific expression of constitutively active Nrf2, which leads to a robust upregulation of Nrf2 transcriptional targets, did not induce or aggravate liver damage in autophagy-competent WT [51] or NEMO^LPC-KO^ mice, respectively (this study). Accordingly, a recent study using transgenic mice with impairment in selective clearance of p62 bodies, but not in bulk autophagy, showed that Nrf2 hyperactivation in vivo did not cause a liver pathology unless when combined with ATG7 ablation [65]. Interestingly, hepatocyte-specific expression of Nrf2ca was shown to provoke higher apoptosis levels after partial hepatectomy [51]. Altogether, these data suggest that whether or not Nrf2 overactivation will promote hepatocyte apoptosis is context-dependent. Particularly in mice with liver-specific autophagy inhibition, perturbations of additional yet-undefined cellular pathways, which are not affected in NEMO-deficient hepatocytes, are required to potentiate the cell death-promoting role of p62 and Nrf2.

It is also worth noting that although Nrf2ca expression did not attenuate liver damage in NEMO^LPC-KO^ mice, feeding with the potent Nrf2 activator BHA did prevent liver damage in these mice [5,49]. Interestingly, BHA was recently reported to inhibit RIPK1 kinase activity independent of its ROS scavenging activity [66]. Considering that the liver damage in NEMO^LPC-KO^ mice is largely RIPK1 kinase activity dependent [4], it is possible that the beneficial effect of BHA in this model is at least in part due to RIPK1 inhibition rather than Nrf2 activation. Yet, a protective effect of BHA as scavenger of ROS produced by other cell types, e.g., immune cells, cannot be excluded.

### 4.3. Defective Autophagy Is Required to Potentiate the Hepatocarcinogenic Role of the p62-Keap1-Nrf2 Axis

The pro-carcinogenic role of the Keap1-Nrf2 pathway is well-documented, as NRF2-activating and KEAP1-inactivating mutations have been identified in a significant percentage of human HCCs [27,28,29]. However, the frequency of NRF2 hyperactivation due to p62 accumulation in the absence of NRF2 and KEAP1 mutations has only recently started being explored [67]. p62 upregulation is observed in most human CLDs that predispose to HCC progression [68], but whether there is a certain threshold or additional molecular requirements for p62 to elicit strong NRF2 activation is unclear. For instance, the conditions when p62 is phosphorylated at S349 (mouse S351), which is situated in its KIR motif and is necessary for KEAP1 sequestration, needs to be further investigated [47]. A study that examined HCCs from a small cohort of patients with different underlying CLD etiologies reported a correlation between activation of the p62-Keap1-Nrf2 pathway and the presence of HCV infection. A proposed explanation for this preference was the HCV-specific inhibition of autophagosome-to-lysosome fusion [53]. On the other hand, Umemura et al. (2016) showed that virus-mediated p62 overexpression alone promotes hepatocarcinogenesis in WT mice. This study postulated a rather universal, autophagy-independent, pro-tumorigenic ability of p62, although the autophagy status in the p62-induced tumors observed after 12 months was not evaluated [44].

The increased tumor burden in NEMO^LPC-KO^ mice that express the p62ΔEx2-5 mutant provide further support for the critical role of p62 in promoting hepatocarcinogenesis. This effect was uncoupled from the level of hepatocellular injury at earlier stages of CLD development that remained unaffected by p62ΔEx2-5 expression. In contrast, the pro-tumorigenic role of p62 in ATG7^Δhepa^ mice cannot be uncoupled from the preceding liver damage, as p62 deficiency ameliorated both events [18,21]. The mechanism how p62ΔEx2-5 promotes hepatocarcinogenesis in NEMO^LPC-KO^ mice could relate to a function that is either preserved or absent in this mutant. As an example of the latter possibility, mice with global p62 deficiency were shown to have increased tumor burden compared to control mice when treated with DEN followed by a high fat diet (HFD) or carbon tetrachloride (CCl_4_) to induce liver injury. This effect was attributed to an inhibitory role of p62 in the activation of hepatic stellate cells, thereby limiting fibrosis-driven hepatocarcinogenesis [55]. Although the detrimental effect of p62 deficiency in NEMO^LPC-KO^ mice precluded assessment of hepatocarcinogenesis in aged mice, the ubiquitous p62ΔEx2-5 expression, which did rescue the early lethality, did not lead to increased levels of α-SMA (fibrosis marker). This suggests that overt activation of HSCs by p62ΔEx2-5 could not explain the increased tumor burden in NEMO^LPC-KO^ p62ΔEx2-5 mice. Moreover, liver lysates from aged NEMO^LPC-KO^ and NEMO^LPC-KO^ p62ΔEx2-5 exhibited no consistent differences in mTORC1 activation and c-MYC expression, which are also known pro-tumorigenic pathways that are regulated by p62 [44,54]. Conversely, stronger Nrf2 activation was the only consistent difference we observed in the livers of NEMO^LPC-KO^ p62ΔEx2-5 mice, and this was coupled to increased levels of p62ΔEx2-5 compared to full-length p62 levels in NEMO^LPC-KO^ mice. Although the reason for the higher p62ΔEx2-5 levels in aged mice is unclear, these findings imply that the p62-Keap1-Nrf2 axis becomes a critical mediator of hepatocarcinogenesis with ageing. These results are in line with a study in DEN-induced hepatocarcinogenesis in rats showing that Nrf2 activation is sustained by *Nrf2* or *Keap1* mutations at pre-neoplastic stages and by p62 accumulation at advanced stages of hepatocarcinogenesis [67].

Our IHC and qRT-PCR analyses in NEMO^LPC-KO^ mice confirmed the prevalence of the p62-Keap1-Nrf2 axis activation, albeit in a lower fraction of liver tumors (10–15%). The reverse correlation between p62 accumulation and the GFP-LC3 signal that we observed in our in situ tumor analysis suggests that mutations leading to autophagy impairment are likely to be a required oncogenic event that can unleash the pro-tumorigenic function of p62 through Nrf2 overactivation and maybe other signaling pathways [63]. This also implies that in advanced HCCs with high p62 levels and Nrf2 signature, disrupting the p62-Keap1 interaction through small molecules [53], applying Nrf2 inhibitors [30] or inducing autophagy with mTOR inhibitors could be beneficial by limiting activation of the p62-Keap1-Nrf2 axis. On the contrary, at precancerous stages of liver pathologies, therapeutic approaches may include Nrf2 activators [30], although considering our data with NEMO^LPC-KO^ Nrf2ca^LPC^ mice, a positive outcome will depend on whether increased oxidative stress is the underlying cause of liver injury. Additional molecular studies are also required to identify pathways that are co-regulated with p62-Keap1-Nrf2 activation. Our qRT-PCR analysis in a limited number of liver tumors provided hints for an inverse correlation between p62-Keap1-Nrf2 activation and expression of the HCC marker Gpc3. Gpc3 is a negative regulator of hedgehog signaling and its low expression or absence has been associated with highly differentiated liver tumors and better survival rate in HCC patients [56]. Conversely, a number of liver tumors observed in ATG5/Tsc1/p62^Δhepa^ mice were strongly expressing Gpc3 [64], suggesting that a putative crosstalk between p62 and Gpc3 could partly explain the enhanced hepatocarcinogenesis in these mice. A more in-depth characterization of the molecular signature associated with p62-KEAP1-NRF2-induced tumors would warrant a better stratification of HCC patients for clinical studies and more targeted treatments.

## 5. Conclusions

In our study, we use a genetic mouse model (NEMO^LPC-KO^) of chronic liver disease and hepatocarcinogenesis, which has no inherent autophagy impairment, to study the role of p62-Keap1-Nrf2 overactivation. We conclude that: (1) Nrf2 hyperactivation without concomitant autophagy impairment and aberrant p62 accumulation does not alter the observed liver injury and tumor burden in NEMO^LPC-KO^ mice; (2) In contrast to its beneficial effect in mice with a hepatic autophagy defect, systemic p62 deficiency leads to perinatal lethality of NEMO^LPC-KO^ mice; (3) Expression of a p62 mutant does not impair survival, but exacerbates hepatocarcinogenesis. The increased tumor burden was associated with aberrant p62 mutant aggregates and Nrf2 overactivation, but not with other protumorigenic functions of p62 through regulation of fibrosis, cMYC expression or mTOR activity.

Our data suggest that autophagy defects due to genetic mutations or ageing are required for the development liver tumors characterized by p62-Keap1-Nrf2 overactivation. This advocates that the use of autophagy activators or inhibitors of the p62-Keap1 interaction or Nrf2-mediated transcription could have a clinical benefit, specifically in patients with p62/Nrf2^high^ (pre)neoplastic lesions.

## Figures and Tables

**Table 1:** The antibodies used for immunoblotting and immunostaining procedures, together with their supplier and product number.

Antibody Name	Supplier	Product Number
p62	Progen	Cat#GP62-C
p-p62(S351)	MBL	Cat#PM074
GAPDH	Novus Biologicals	Cat#NB300-221
LC3B	Nanotools	Cat# 0231-100/LC3-5F10
LC3B	Novusbio	Cat#NB600-1384
p-S6	Cell Signaling	Cat#5364
S6	Cell Signaling	Cat#2217
p-S6K	Cell Signaling	Cat#9205
p-AMPK	Cell Signaling	Cat#2535
AMPK	Cell Signaling	Cat#2532
α-Tubulin	Sigma- Aldrich	Cat#T6074
Actin	Santa Cruz	Cat#sc-1616
NEMO	Homemade	[4]
IKK2	Imgenex	Cat#IMG-129A
ATG16L	MBL	Cat#M150-3
Cleaved Casp-3	Cell Signaling	Cat#9661
Ki-67	DAKO	Cat#M724901
PCNA	Santa Cruz	Cat#sc-56
α-SMA	Sigma-Aldrich	Cat#A2547
F4/80	Bio-Rad	Cat#MCA497GA
Ubiquitin	Enzo	Cat#PW8810/0500
FADD	Upstate	Cat#05-486
GFP	Abcam	Cat#Ab290
Nqo1	Elabscience	Cat#E-AB-16011

## Data Availability

The data presented in this study are available in this article.

## Supplementary Materials

The following supporting information can be downloaded at: https://www.mdpi.com/article/10.3390/cancers14102436/s1, Figure S1: Starvation can efficiently induce autophagosome formation and p62 aggregate clearance in hepatocytes of NEMO^LPC-KO^ mice; Figure S2: IKK1 deficiency, IKK2 deficiency or IKK2ca expression does not affect autophagosome formation in primary hepatocytes; Figure S3: Different molecular pathways drive the liver pathologies in ATG16L1^LPC-KO^ and NEMO^LPC-KO^ mice; Figure S4: Late-onset obesity and fatty liver observed in p62^KO^ mice are strongly prevented in p62^ΔEx2-5^ mice; Figure S5: Uncropped scans of immunoblots presented in the manuscript.

## References

[1] Guicciardi M.E., Malhi H., Mott J.L., Gores G.J. (2013). Apoptosis and necrosis in the liver. Compr. Physiol..

[2] Schwabe R.F., Luedde T. (2018). Apoptosis and necroptosis in the liver: A matter of life and death. Nat. Rev. Gastroenterol. Hepatol..

[3] Kondylis V., Pasparakis M. (2019). RIP Kinases in Liver Cell Death, Inflammation and Cancer. Trends Mol. Med..

[4] Kondylis V., Polykratis A., Ehlken H., Ochoa-Callejero L., Straub B.K., Krishna-Subramanian S., Van T.M., Curth H.M., Heise N., Weih F. (2015). NEMO Prevents Steatohepatitis and Hepatocellular Carcinoma by Inhibiting RIPK1 Kinase Activity-Mediated Hepatocyte Apoptosis. Cancer Cell.

[5] Luedde T., Beraza N., Kotsikoris V., van Loo G., Nenci A., De Vos R., Roskams T., Trautwein C., Pasparakis M. (2007). Deletion of NEMO/IKKgamma in liver parenchymal cells causes steatohepatitis and hepatocellular carcinoma. Cancer Cell.

[6] Ting A.T., Bertrand M.J.M. (2016). More to Life than NF-kappaB in TNFR1 Signaling. Trends Immunol..

[7] Dondelinger Y., Jouan-Lanhouet S., Divert T., Theatre E., Bertin J., Gough P.J., Giansanti P., Heck A.J., Dejardin E., Vandenabeele P. (2015). NF-kappaB-Independent Role of IKKalpha/IKKbeta in Preventing RIPK1 Kinase-Dependent Apoptotic and Necroptotic Cell Death during TNF Signaling. Mol. Cell.

[8] Ehlken H., Krishna-Subramanian S., Ochoa-Callejero L., Kondylis V., Nadi N.E., Straub B.K., Schirmacher P., Walczak H., Kollias G., Pasparakis M. (2014). Death receptor-independent FADD signalling triggers hepatitis and hepatocellular carcinoma in mice with liver parenchymal cell-specific NEMO knockout. Cell Death Differ..

[9] Criollo A., Senovilla L., Authier H., Maiuri M.C., Morselli E., Vitale I., Kepp O., Tasdemir E., Galluzzi L., Shen S. (2010). The IKK complex contributes to the induction of autophagy. EMBO J..

[10] Comb W.C., Cogswell P., Sitcheran R., Baldwin A.S. (2011). IKK-dependent, NF-kappaB-independent control of autophagic gene expression. Oncogene.

[11] Verzella D., Pescatore A., Capece D., Vecchiotti D., Ursini M.V., Franzoso G., Alesse E., Zazzeroni F. (2020). Life, death, and autophagy in cancer: NF-kappaB turns up everywhere. Cell Death Dis..

[12] Dan H.C., Cooper M.J., Cogswell P.C., Duncan J.A., Ting J.P., Baldwin A.S. (2008). Akt-dependent regulation of NF-{kappa}B is controlled by mTOR and Raptor in association with IKK. Genes Dev..

[13] Inokuchi-Shimizu S., Park E.J., Roh Y.S., Yang L., Zhang B., Song J., Liang S., Pimienta M., Taniguchi K., Wu X. (2014). TAK1-mediated autophagy and fatty acid oxidation prevent hepatosteatosis and tumorigenesis. J. Clin. Investig..

[14] Herrero-Martin G., Hoyer-Hansen M., Garcia-Garcia C., Fumarola C., Farkas T., Lopez-Rivas A., Jaattela M. (2009). TAK1 activates AMPK-dependent cytoprotective autophagy in TRAIL-treated epithelial cells. EMBO J..

[15] Li N., Wu X., Holzer R.G., Lee J.H., Todoric J., Park E.J., Ogata H., Gukovskaya A.S., Gukovsky I., Pizzo D.P. (2013). Loss of acinar cell IKKalpha triggers spontaneous pancreatitis in mice. J. Clin. Investig..

[16] Allaire M., Rautou P.E., Codogno P., Lotersztajn S. (2019). Autophagy in liver diseases: Time for translation?. J. Hepatol..

[17] Czaja M.J., Ding W.X., Donohue T.M., Friedman S.L., Kim J.S., Komatsu M., Lemasters J.J., Lemoine A., Lin J.D., Ou J.H. (2013). Functions of autophagy in normal and diseased liver. Autophagy.

[18] Takamura A., Komatsu M., Hara T., Sakamoto A., Kishi C., Waguri S., Eishi Y., Hino O., Tanaka K., Mizushima N. (2011). Autophagy-deficient mice develop multiple liver tumors. Genes Dev..

[19] Inami Y., Waguri S., Sakamoto A., Kouno T., Nakada K., Hino O., Watanabe S., Ando J., Iwadate M., Yamamoto M. (2011). Persistent activation of Nrf2 through p62 in hepatocellular carcinoma cells. J. Cell Biol..

[20] Ni H.M., Woolbright B.L., Williams J., Copple B., Cui W., Luyendyk J.P., Jaeschke H., Ding W.X. (2014). Nrf2 promotes the development of fibrosis and tumorigenesis in mice with defective hepatic autophagy. J. Hepatol..

[21] Komatsu M., Waguri S., Koike M., Sou Y.S., Ueno T., Hara T., Mizushima N., Iwata J., Ezaki J., Murata S. (2007). Homeostatic levels of p62 control cytoplasmic inclusion body formation in autophagy-deficient mice. Cell.

[22] Strnad P., Stumptner C., Zatloukal K., Denk H. (2008). Intermediate filament cytoskeleton of the liver in health and disease. Histochem. Cell Biol..

[23] Sanchez-Martin P., Komatsu M. (2018). p62/SQSTM1—Steering the cell through health and disease. J. Cell Sci..

[24] Katsuragi Y., Ichimura Y., Komatsu M. (2015). p62/SQSTM1 functions as a signaling hub and an autophagy adaptor. FEBS J..

[25] Moscat J., Karin M., Diaz-Meco M.T. (2016). p62 in Cancer: Signaling Adaptor Beyond Autophagy. Cell.

[26] Komatsu M., Kurokawa H., Waguri S., Taguchi K., Kobayashi A., Ichimura Y., Sou Y.S., Ueno I., Sakamoto A., Tong K.I. (2010). The selective autophagy substrate p62 activates the stress responsive transcription factor Nrf2 through inactivation of Keap1. Nat. Cell Biol..

[27] Guichard C., Amaddeo G., Imbeaud S., Ladeiro Y., Pelletier L., Maad I.B., Calderaro J., Bioulac-Sage P., Letexier M., Degos F. (2012). Integrated analysis of somatic mutations and focal copy-number changes identifies key genes and pathways in hepatocellular carcinoma. Nat. Genet..

[28] Schulze K., Imbeaud S., Letouze E., Alexandrov L.B., Calderaro J., Rebouissou S., Couchy G., Meiller C., Shinde J., Soysouvanh F. (2015). Exome sequencing of hepatocellular carcinomas identifies new mutational signatures and potential therapeutic targets. Nat. Genet..

[29] Shibata T., Aburatani H. (2014). Exploration of liver cancer genomes. Nat. Rev. Gastroenterol. Hepatol..

[30] Orru C., Giordano S., Columbano A. (2020). Nrf2 in Neoplastic and Non-Neoplastic Liver Diseases. Cancers.

[31] Schmidt-Supprian M., Bloch W., Courtois G., Addicks K., Israel A., Rajewsky K., Pasparakis M. (2000). NEMO/IKK gamma-deficient mice model incontinentia pigmenti. Mol. Cell.

[32] Pasparakis M., Courtois G., Hafner M., Schmidt-Supprian M., Nenci A., Toksoy A., Krampert M., Goebeler M., Gillitzer R., Israel A. (2002). TNF-mediated inflammatory skin disease in mice with epidermis-specific deletion of IKK2. Nature.

[33] Gareus R., Huth M., Breiden B., Nenci A., Rosch N., Haase I., Bloch W., Sandhoff K., Pasparakis M. (2007). Normal epidermal differentiation but impaired skin-barrier formation upon keratinocyte-restricted IKK1 ablation. Nat. Cell Biol..

[34] Sasaki Y., Derudder E., Hobeika E., Pelanda R., Reth M., Rajewsky K., Schmidt-Supprian M. (2006). Canonical NF-kappaB activity, dispensable for B cell development, replaces BAFF-receptor signals and promotes B cell proliferation upon activation. Immunity.

[35] Adolph T.E., Tomczak M.F., Niederreiter L., Ko H.J., Bock J., Martinez-Naves E., Glickman J.N., Tschurtschenthaler M., Hartwig J., Hosomi S. (2013). Paneth cells as a site of origin for intestinal inflammation. Nature.

[36] Rodriguez A., Duran A., Selloum M., Champy M.F., Diez-Guerra F.J., Flores J.M., Serrano M., Auwerx J., Diaz-Meco M.T., Moscat J. (2006). Mature-onset obesity and insulin resistance in mice deficient in the signaling adapter p62. Cell Metab..

[37] Skarnes W.C., Rosen B., West A.P., Koutsourakis M., Bushell W., Iyer V., Mujica A.O., Thomas M., Harrow J., Cox T. (2011). A conditional knockout resource for the genome-wide study of mouse gene function. Nature.

[38] Mizushima N., Yamamoto A., Matsui M., Yoshimori T., Ohsumi Y. (2004). In vivo analysis of autophagy in response to nutrient starvation using transgenic mice expressing a fluorescent autophagosome marker. Mol. Biol. Cell.

[39] Schafer M., Farwanah H., Willrodt A.H., Huebner A.J., Sandhoff K., Roop D., Hohl D., Bloch W., Werner S. (2012). Nrf2 links epidermal barrier function with antioxidant defense. EMBO Mol. Med..

[40] Kellendonk C., Opherk C., Anlag K., Schutz G., Tronche F. (2000). Hepatocyte-specific expression of Cre recombinase. Genesis.

[41] Ehlken H., Kondylis V., Heinrichsdorff J., Ochoa-Callejero L., Roskams T., Pasparakis M. (2011). Hepatocyte IKK2 protects Mdr2-/- mice from chronic liver failure. PLoS ONE.

[42] Schneider C.A., Rasband W.S., Eliceiri K.W. (2012). NIH Image to ImageJ: 25 years of image analysis. Nat. Methods.

[43] Menon S., Yecies J.L., Zhang H.H., Howell J.J., Nicholatos J., Harputlugil E., Bronson R.T., Kwiatkowski D.J., Manning B.D. (2012). Chronic activation of mTOR complex 1 is sufficient to cause hepatocellular carcinoma in mice. Sci. Signal..

[44] Umemura A., He F., Taniguchi K., Nakagawa H., Yamachika S., Font-Burgada J., Zhong Z., Subramaniam S., Raghunandan S., Duran A. (2016). p62, Upregulated during Preneoplasia, Induces Hepatocellular Carcinogenesis by Maintaining Survival of Stressed HCC-Initiating Cells. Cancer Cell.

[45] Singh R., Kaushik S., Wang Y., Xiang Y., Novak I., Komatsu M., Tanaka K., Cuervo A.M., Czaja M.J. (2009). Autophagy regulates lipid metabolism. Nature.

[46] Ling J., Kang Y., Zhao R., Xia Q., Lee D.F., Chang Z., Li J., Peng B., Fleming J.B., Wang H. (2012). KrasG12D-induced IKK2/beta/NF-kappaB activation by IL-1alpha and p62 feedforward loops is required for development of pancreatic ductal adenocarcinoma. Cancer Cell.

[47] Ichimura Y., Waguri S., Sou Y.S., Kageyama S., Hasegawa J., Ishimura R., Saito T., Yang Y., Kouno T., Fukutomi T. (2013). Phosphorylation of p62 activates the Keap1-Nrf2 pathway during selective autophagy. Mol. Cell.

[48] Krishna-Subramanian S., Singer S., Armaka M., Banales J.M., Holzer K., Schirmacher P., Walczak H., Kollias G., Pasparakis M., Kondylis V. (2019). RIPK1 and death receptor signaling drive biliary damage and early liver tumorigenesis in mice with chronic hepatobiliary injury. Cell Death Differ..

[49] Huang Y., Li W., Su Z.Y., Kong A.N. (2015). The complexity of the Nrf2 pathway: Beyond the antioxidant response. J. Nutr. Biochem..

[50] Taguchi K., Fujikawa N., Komatsu M., Ishii T., Unno M., Akaike T., Motohashi H., Yamamoto M. (2012). Keap1 degradation by autophagy for the maintenance of redox homeostasis. Proc. Natl. Acad. Sci. USA.

[51] Kohler U.A., Kurinna S., Schwitter D., Marti A., Schafer M., Hellerbrand C., Speicher T., Werner S. (2014). Activated Nrf2 impairs liver regeneration in mice by activation of genes involved in cell-cycle control and apoptosis. Hepatology.

[52] Jain A., Lamark T., Sjottem E., Larsen K.B., Awuh J.A., Overvatn A., McMahon M., Hayes J.D., Johansen T. (2010). p62/SQSTM1 is a target gene for transcription factor NRF2 and creates a positive feedback loop by inducing antioxidant response element-driven gene transcription. J. Biol. Chem..

[53] Saito T., Ichimura Y., Taguchi K., Suzuki T., Mizushima T., Takagi K., Hirose Y., Nagahashi M., Iso T., Fukutomi T. (2016). p62/Sqstm1 promotes malignancy of HCV-positive hepatocellular carcinoma through Nrf2-dependent metabolic reprogramming. Nat. Commun..

[54] Duran A., Amanchy R., Linares J.F., Joshi J., Abu-Baker S., Porollo A., Hansen M., Moscat J., Diaz-Meco M.T. (2011). p62 is a key regulator of nutrient sensing in the mTORC1 pathway. Mol. Cell.

[55] Duran A., Hernandez E.D., Reina-Campos M., Castilla E.A., Subramaniam S., Raghunandan S., Roberts L.R., Kisseleva T., Karin M., Diaz-Meco M.T. (2016). p62/SQSTM1 by Binding to Vitamin D Receptor Inhibits Hepatic Stellate Cell Activity, Fibrosis, and Liver Cancer. Cancer Cell.

[56] Guo M., Zhang H., Zheng J., Liu Y. (2020). Glypican-3: A New Target for Diagnosis and Treatment of Hepatocellular Carcinoma. J. Cancer.

[57] Amaravadi R., Kimmelman A.C., White E. (2016). Recent insights into the function of autophagy in cancer. Genes Dev..

[58] Luedde T., Heinrichsdorff J., de Lorenzi R., De Vos R., Roskams T., Pasparakis M. (2008). IKK1 and IKK2 cooperate to maintain bile duct integrity in the liver. Proc. Natl. Acad. Sci. USA.

[59] Criollo A., Niso-Santano M., Malik S.A., Michaud M., Morselli E., Marino G., Lachkar S., Arkhipenko A.V., Harper F., Pierron G. (2011). Inhibition of autophagy by TAB2 and TAB3. EMBO J..

[60] Tusco R., Jacomin A.C., Jain A., Penman B.S., Larsen K.B., Johansen T., Nezis I.P. (2017). Kenny mediates selective autophagic degradation of the IKK complex to control innate immune responses. Nat. Commun..

[61] Yang S., Qiang L., Sample A., Shah P., He Y.Y. (2017). NF-kappaB Signaling Activation Induced by Chloroquine Requires Autophagosome, p62 Protein, and c-Jun N-terminal Kinase (JNK) Signaling and Promotes Tumor Cell Resistance. J. Biol. Chem..

[62] Duran A., Linares J.F., Galvez A.S., Wikenheiser K., Flores J.M., Diaz-Meco M.T., Moscat J. (2008). The signaling adaptor p62 is an important NF-kappaB mediator in tumorigenesis. Cancer Cell.

[63] Mathew R., Karp C.M., Beaudoin B., Vuong N., Chen G., Chen H.Y., Bray K., Reddy A., Bhanot G., Gelinas C. (2009). Autophagy suppresses tumorigenesis through elimination of p62. Cell.

[64] Chao X., Wang S., Fulte S., Ma X., Ahamed F., Cui W., Liu Z., Rulicke T., Zatloukal K., Zong W.X. (2022). Hepatocytic p62 suppresses ductular reaction and tumorigenesis in mouse livers with mTORC1 activation and defective autophagy. J. Hepatol..

[65] Kageyama S., Gudmundsson S.R., Sou Y.S., Ichimura Y., Tamura N., Kazuno S., Ueno T., Miura Y., Noshiro D., Abe M. (2021). p62/SQSTM1-droplet serves as a platform for autophagosome formation and anti-oxidative stress response. Nat. Commun..

[66] Delanghe T., Huyghe J., Lee S., Priem D., Van Coillie S., Gilbert B., Choi S.M., Vandenabeele P., Degterev A., Cuny G.D. (2021). Antioxidant and food additive BHA prevents TNF cytotoxicity by acting as a direct RIPK1 inhibitor. Cell Death Dis..

[67] Orru C., Perra A., Kowalik M.A., Rizzolio S., Puliga E., Cabras L., Giordano S., Columbano A. (2020). Distinct Mechanisms Are Responsible for Nrf2-Keap1 Pathway Activation at Different Stages of Rat Hepatocarcinogenesis. Cancers.

[68] Denk H., Stumptner C., Fuchsbichler A., Muller T., Farr G., Muller W., Terracciano L., Zatloukal K. (2006). Are the Mallory bodies and intracellular hyaline bodies in neoplastic and non-neoplastic hepatocytes related?. J. Pathol..

