# Supplementary material for: p62 Promotes Survival and Hepatocarcinogenesis in Mice with Liver-Specific NEMO Ablation
Source: Cancers (Basel). 2022 May 15;14(10):2436. doi: 10.3390/cancers14102436 (PMC9139637; doi:10.3390/cancers14102436)
Supplement: Supplementary file 1 [file cancers-14-02436-s001.zip › cancers-1698084-supplementary.pdf]

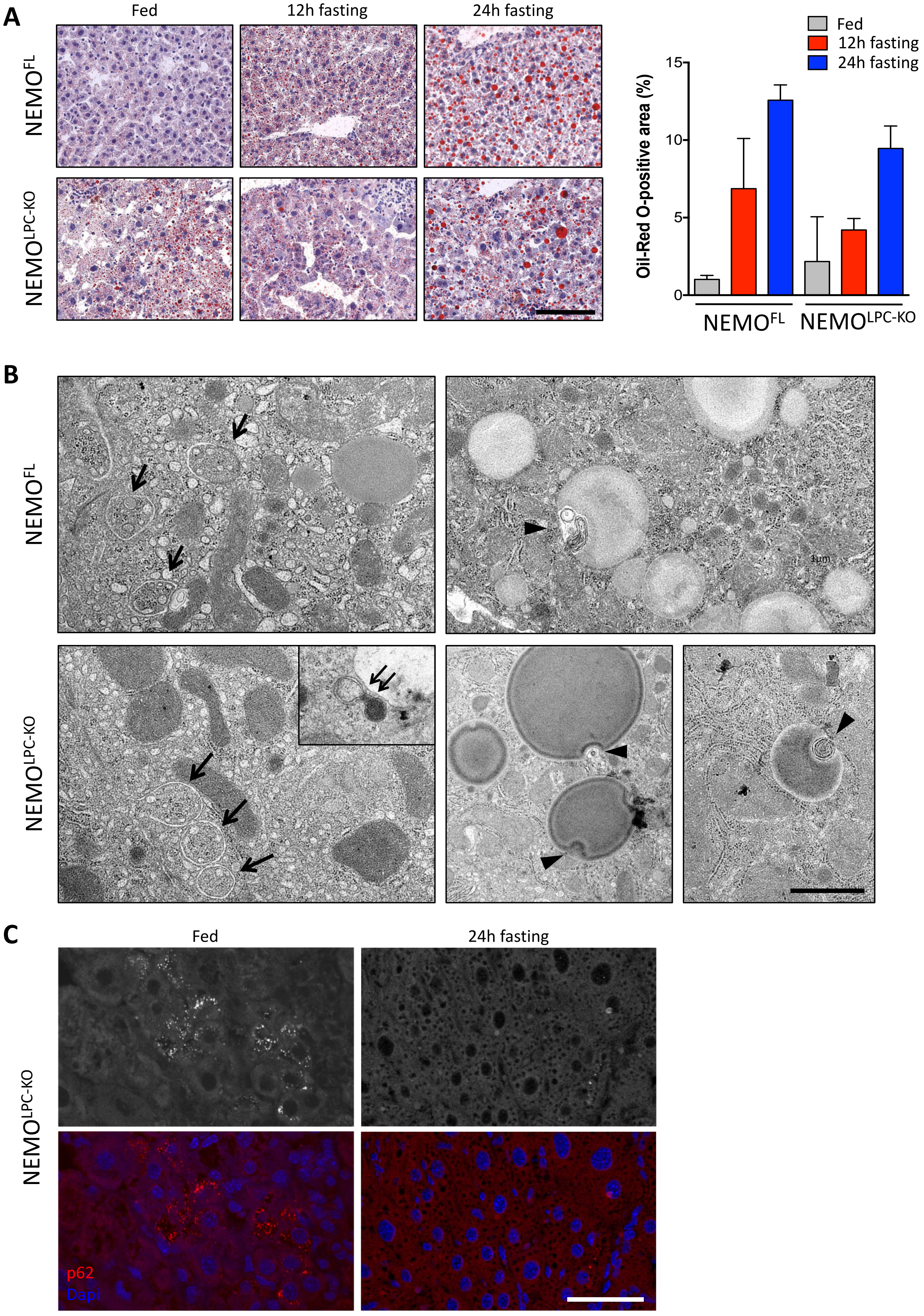
A
Fed
12h fasting
24h fasting
NEMOFL
NEMOLPC-KO
Oil-Red O-positive area (%)
15
10
5
0
Fed
12h fasting
24h fasting
NEMOFL
NEMOLPC-KO
B
NEMOFL
NEMOLPC-KO
C
Fed
24h fasting
NEMOLPC-KO
p62
Dapi

Figure S1

Figure S1: **Starvation can efficiently induce autophagosome formation and p62 aggregate clearance in hepatocytes of NEMO^{LPC-KO} mice.** (A) Representative images of Oil Red O-stained liver sections from 8-week-old NEMO^{LPC-KO} mice and age-matched controls fed or fasted for 12 or 24 h. Image quantification is shown on the right. Graphs depict mean ±sd (n=3). (B) Representative electron microscopy pictures showing autophagosomes (arrows), autolysosomes (double arrow) and lipophagy profiles (arrowheads) in hepatocytes of both NEMO^{LPC-KO} and control mice. (C) Representative pictures of liver sections immunostained for p62 from 8-week-old NEMO^{LPC-KO} mice fed or fasted for 24h. DAPI is used to stain the nuclei. Bars: 100μm (A), 1μm (B), 50μm (C).

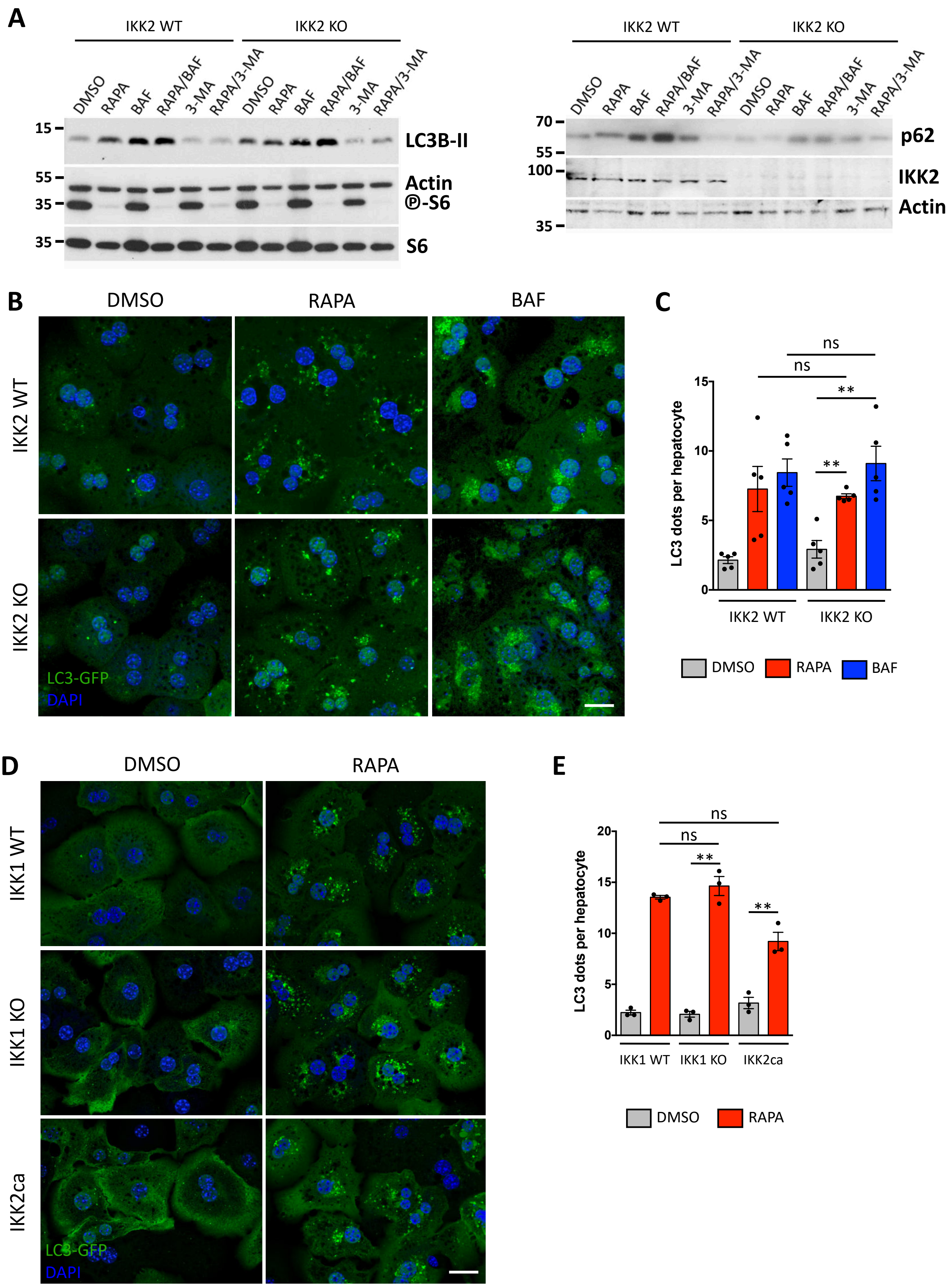
A
IKK2 WT
IKK2 KO
DMSO
RAPA
BAF
RAPA/BAF
3-MA
RAPA/3-MA
15
55
35
35
LC3B-II
Actin
Ⓟ-S6
S6
70
55
100
35
p62
IKK2
Actin
B
DMSO
RAPA
BAF
IKK2 WT
IKK2 KO
LC3-GFP
DAPI
C
LC3 dots per hepatocyte
ns
ns
**
**
15
10
5
0
IKK2 WT
IKK2 KO
DMSO
RAPA
BAF
D
DMSO
RAPA
IKK1 WT
IKK1 KO
IKK2ca
LC3-GFP
DAPI
E
LC3 dots per hepatocyte
ns
ns
**
**
20
15
10
5
0
IKK1 WT
IKK1 KO
IKK2ca
DMSO
RAPA

Figure S2

<u>Figure S2:</u> **IKK1 deficiency, IKK2 deficiency or IKK2ca expression does not affect autophagosome formation in primary hepatocytes.** (A) Immunoblot analysis of the indicated proteins in total lysates from WT and IKK2 KO primary hepatocytes treated for 6 h with drugs that affect autophagic flux. Actin is used as loading control. Representative results from 3 independent experiments are shown. (B-C) Representative pictures (B) and quantification (C) of IF staining for LC3 in WT and IKK2 KO hepatocytes upon treatment with Baf-A1 or Rapamycin for 2 h (n=5). (D-E) Representative pictures (D) and quantification (E) of IF staining of LC3 in WT, IKK1 KO and IKK2ca-expressing hepatocytes upon treatment with Baf-A1 or Rapamycin for 2 h (n=3). In B and D, DAPI is used to stain the nuclei. Graphs depict mean ±sem. Bars: 20µm.

A

$Nemo^{FL/Y}$; $AfpCre^{tg/wt}$; $p62^{ko/ko}$ x $Nemo^{FL/FL}$; $AfpCre^{wt/wt}$; $p62^{ko/ko}$	Expected	Observed	Number of mice born
$Nemo^{FL}$; $AfpCre^{tg/wt}$; $p62^{ko/ko}$	50%	**26.1%**	6
$Nemo^{FL}$; $AfpCre^{wt/wt}$; $p62^{ko/ko}$	50%	73.9%	17

$Nemo^{FL/Y}$; $AfpCre^{tg/wt}$; $p62^{wt/ko}$ x $Nemo^{FL/FL}$; $AfpCre^{wt/wt}$; $p62^{wt/ko}$	Expected	Observed	Number of mice born
$Nemo^{FL}$; $AfpCre^{tg/wt}$; $p62^{ko/ko}$	25%	**4.5%**	3
$Nemo^{FL}$; $AfpCre^{wt/wt}$; $p62^{ko/ko}$	25%	28.4%	19
$Nemo^{FL}$; $AfpCre^{tg/wt}$; $p62^{wt/ko}$	25%	29.8%	20
$Nemo^{FL}$; $AfpCre^{wt/wt}$; $p62^{wt/ko}$	25%	37.3%	25

B

$NEMO^{LPC-KO}$ $p62^{KO}$ survivors	Age during death
3	< 3 days
2	< 3 weeks
3	5-6 weeks
1	21 weeks
1	50 weeks

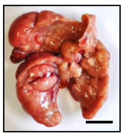

Figure S3

<u>Figure S3:</u> **Different molecular pathways drive the liver pathologies in ATG16L1^{LPC-KO} and NEMO^{LPC-KO} mice.** (A) Immunoblot analysis in total liver lysates from 8-week-old mice of the indicated genotypes. GAPDH is used as loading control. (B) Serum ALT levels in 8-week-old mice with the indicated genotypes. Horizontal lines indicate mean values (n=8-31 mice). (C) Representative images of liver sections from 8-week-old mice with the indicated genotypes after immunostaining for specific disease markers. (D) Quantification of the depicted markers in C. Graphs show mean ±sem (n=3-8 mice per genotype). (E) Table showing the expected Mendelian frequency and the observed frequency and number of born pups with the indicated genotypes. (F) Table showing the time of spontaneous death of the NEMO^{LPC-KO} p62^{KO} mice that were born and liver picture of the single mouse with this genotype that survived to the age of 1 year. Bars: (C) 200 μm; (F) 1 cm.

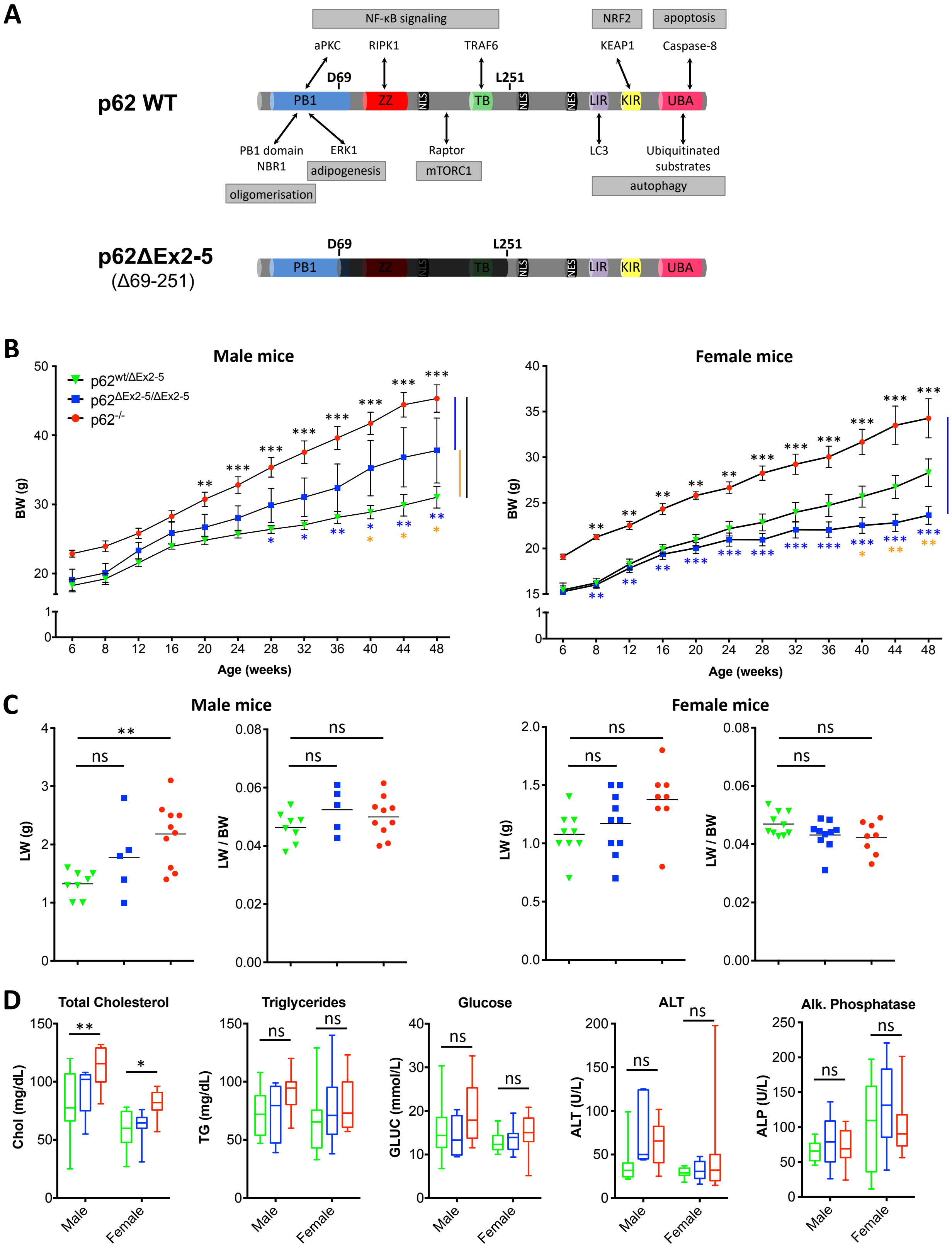

Figure S4

Figure S4: **Late-onset obesity and fatty liver observed in p62^{KO} mice are strongly prevented in p62$^{\Delta Ex2\text{-}5}$ mice.** (A) Schematic representation of structural domains in wildtype p62/SQSTM1 and p62ΔEx2-5 mutant along with previously described functional interactions. p62ΔEx2-5 mutant lacks the part of the protein between Asp69 (D69) and Leu251 (L251), which is shaded in black. (B) Body weight (BW) development in control (p62$^{wt/\Delta Ex2\text{-}5}$), p62$^{\Delta Ex2\text{-}5}$ and p62^{KO} male and female mice up to

48 weeks of age (n=8, 5, 10 and n=10, 10, 8 for male and female, respectively). Pairwise statistical significance is indicated by the different colors. (C) Liver weight (LW) and LW/BW ratio of the same mice as in B. (D) Serum levels of total cholesterol, triglycerides, non-fasting glucose, ALT and Alkaline phosphatase in 48-week-old mice described in B.

Figure S5

Figure 1C

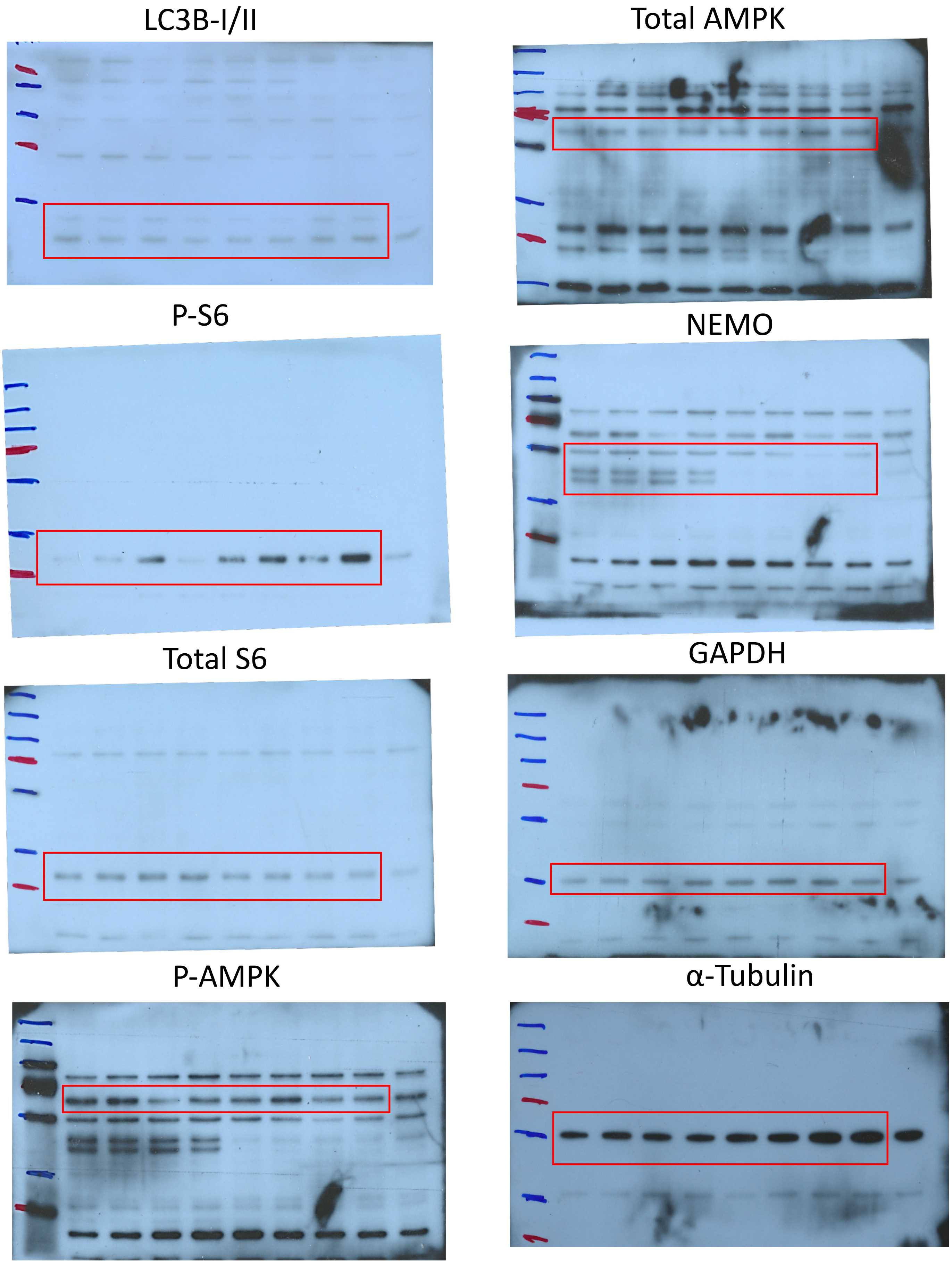

Figure 1D

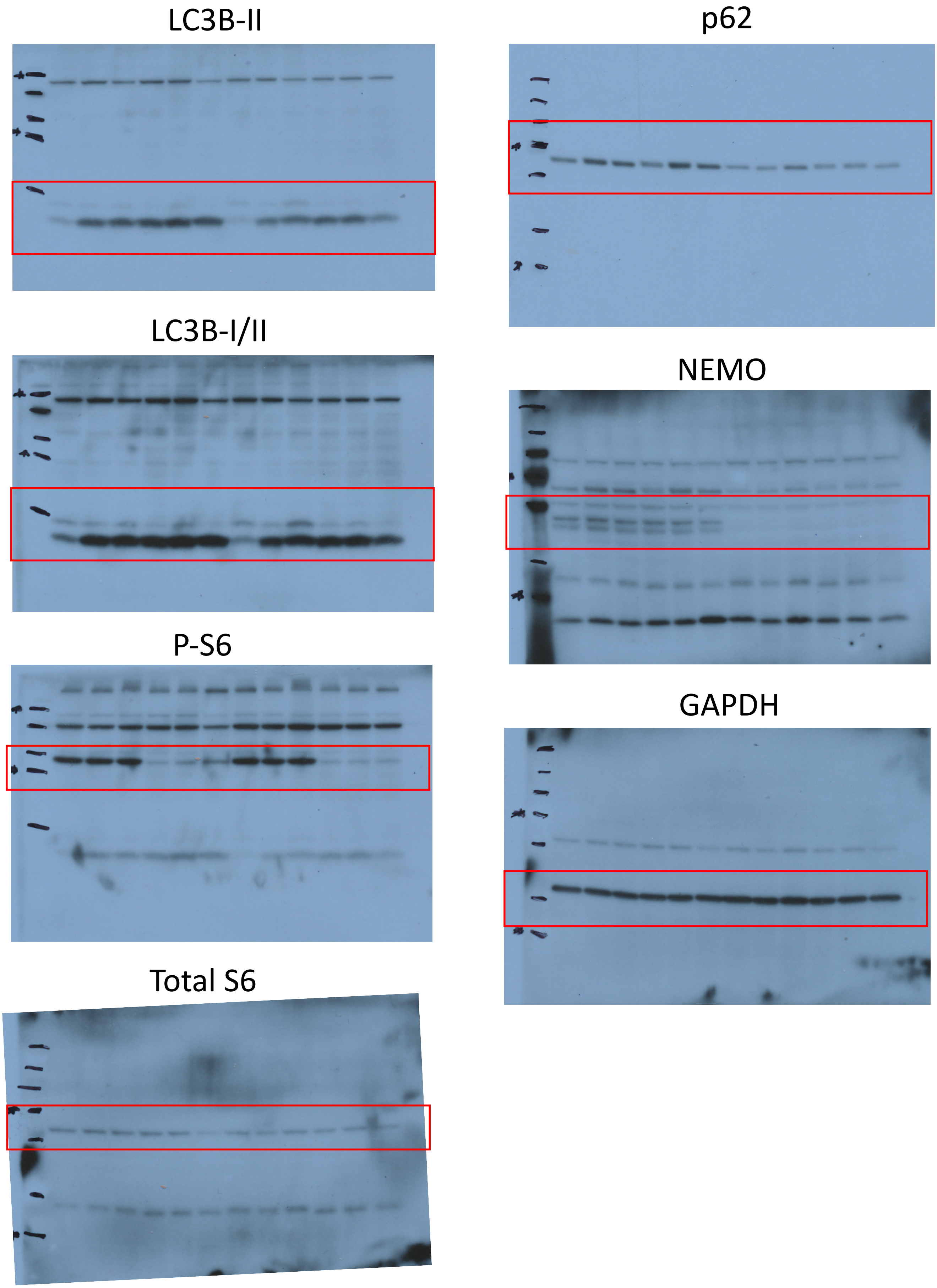

Figure 1E

LC3B-II

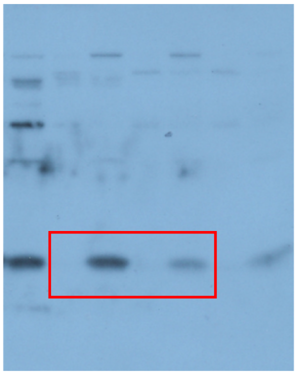

P-S6

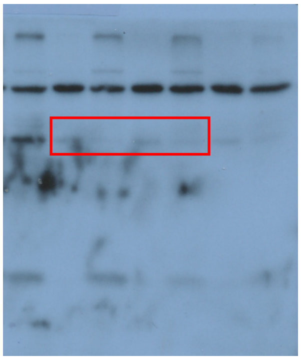

P-AMPK

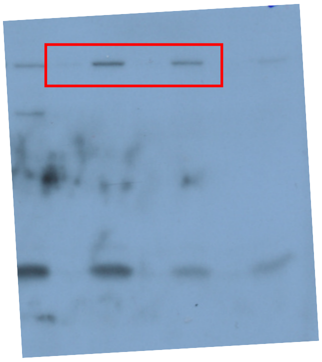

NEMO

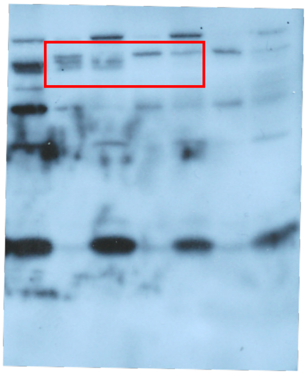

α-Tubulin/GAPDH

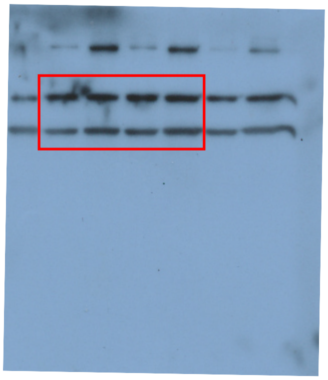

Figure 2A

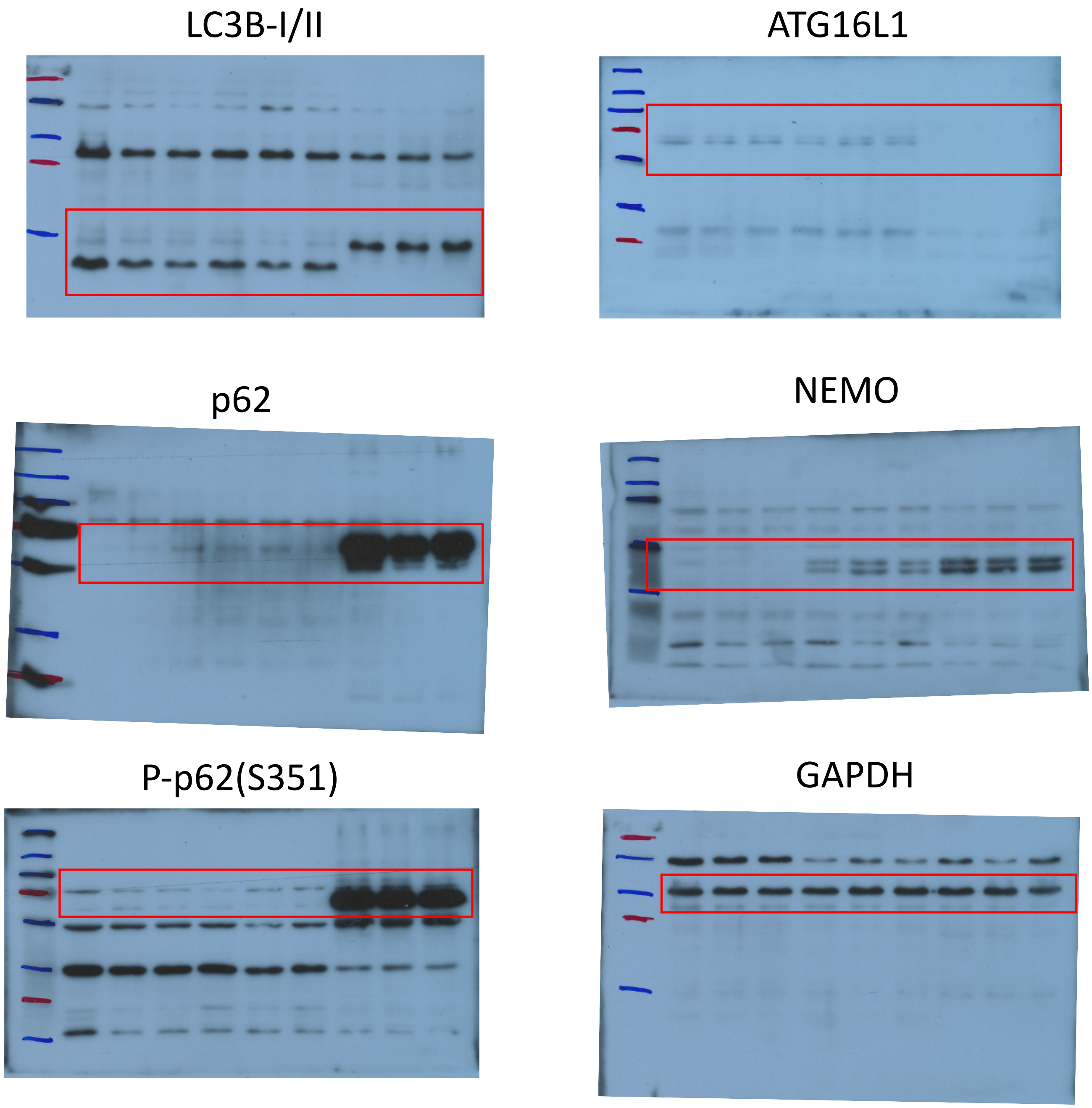

Figure 4A

p62

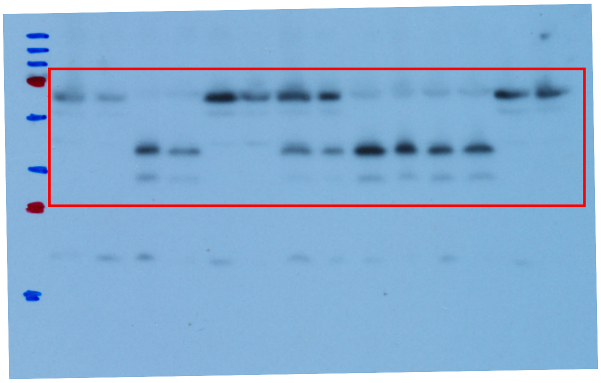

NEMO

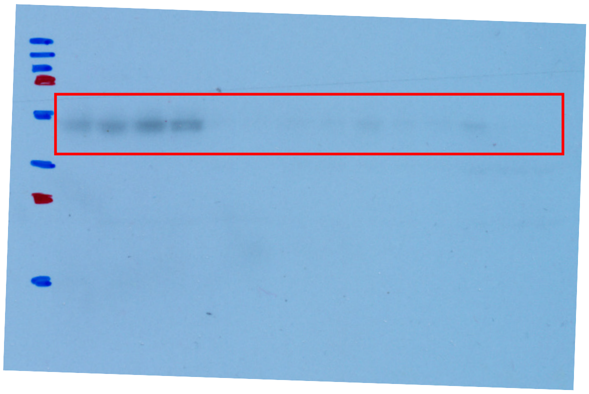

NQO1

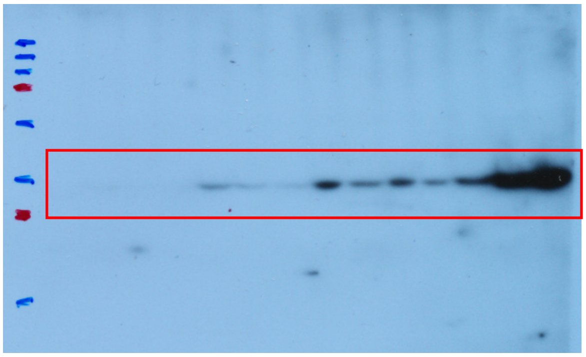

α-Tubulin

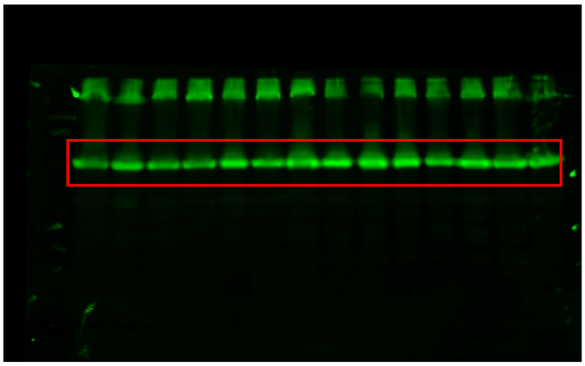

Figure 7B

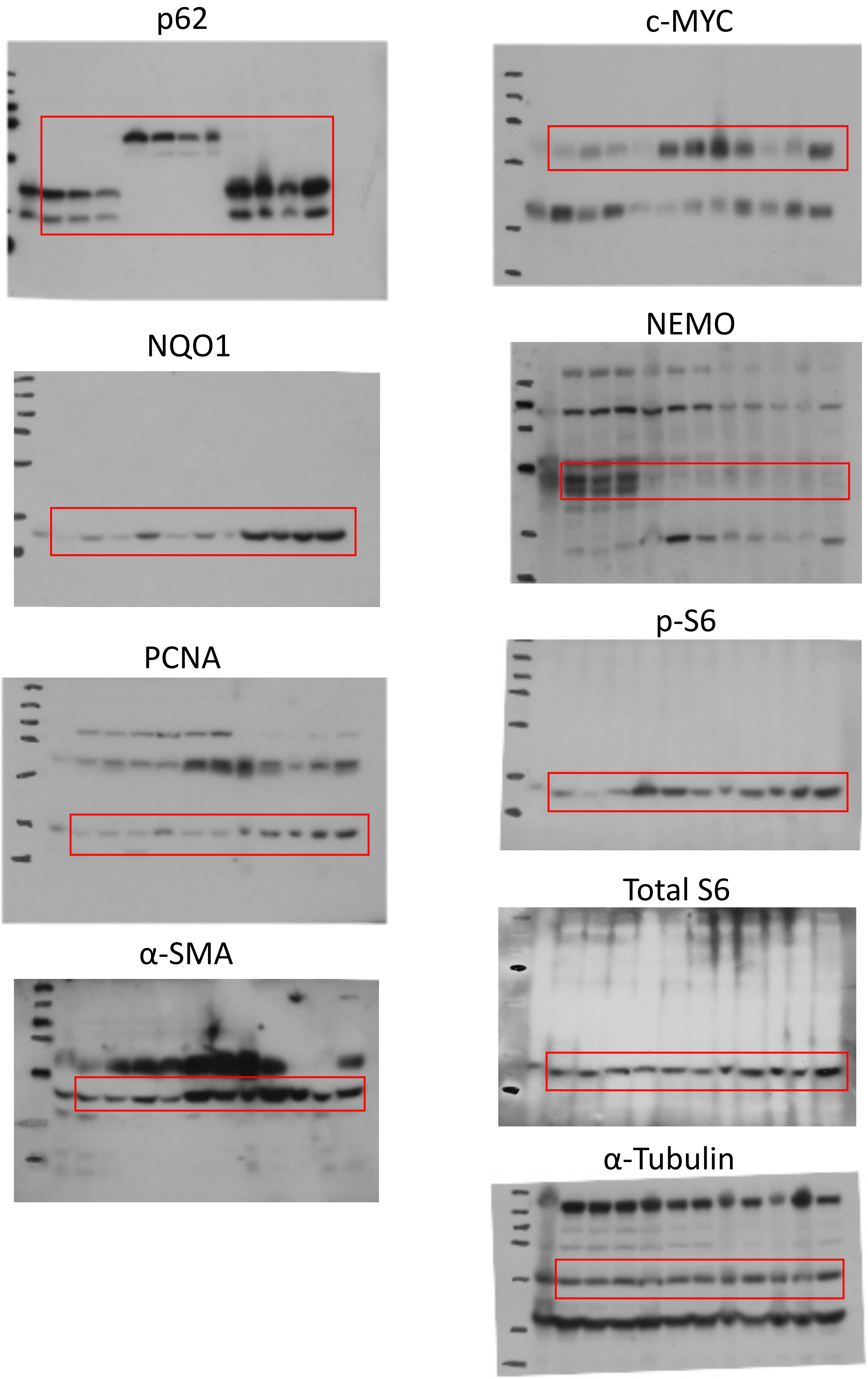

Figure S2A

LC3B-II

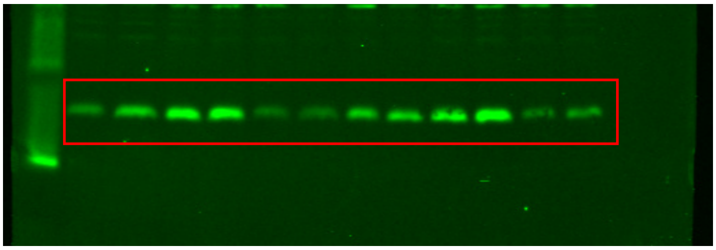

p62

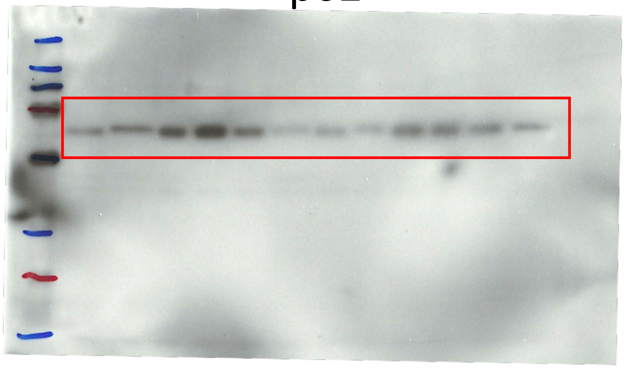

Actin/p-S6

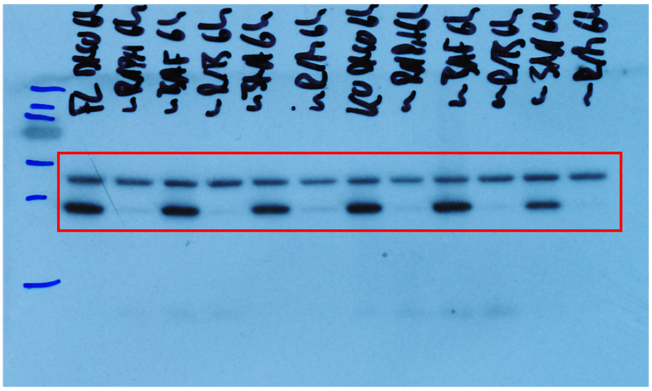

IKK2

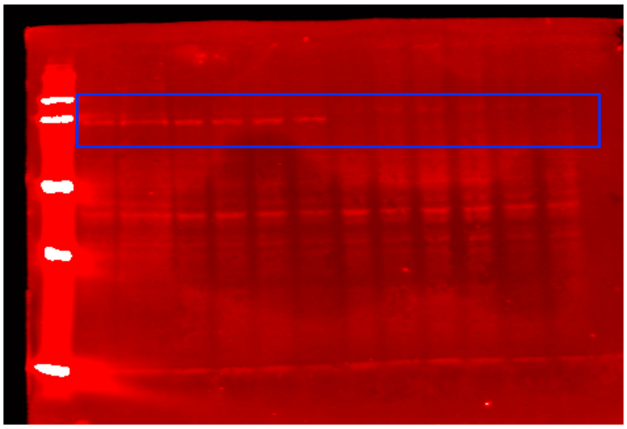

Total S6

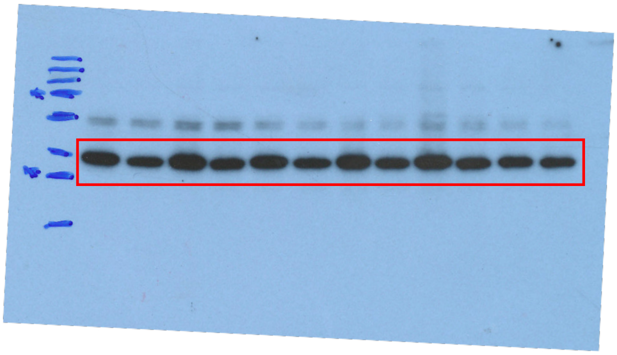

Actin

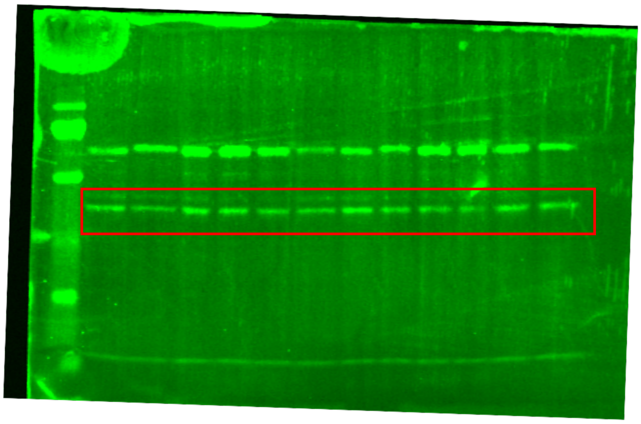

Figure S5. **Uncropped scans of immunoblots presented in the manuscript.**
